# Applying the illness-death model to estimate the incidence and remission of severe anxiety and depressive symptoms in the German National Cohort (NAKO)

**DOI:** 10.1192/j.eurpsy.2026.10154

**Published:** 2026-01-29

**Authors:** Chisato Ito, Bernhard T. Baune, Klaus Berger, The German National Cohort (NAKO) Investigators, Tobias Kurth, Ralph Brinks

**Affiliations:** 1 Institute of Public Health, Charité – Universitätsmedizin Berlin, Berlin, Germany; 2 Department of Psychiatry, University of Münster, Münster, Germany; 3 Department of Psychiatry, Melbourne Medical School, University of Melbourne, Melbourne, Australia; 4 Florey Institute of Neuroscience and Mental Health University of Melbourne, Melbourne, Australia; 5 Institute of Epidemiology and Social Medicine, University of Münster, Münster, Germany; 6 Chair for Medical Biometry and Epidemiology, Faculty of Health/School of Medicine, Witten/Herdecke University, Witten, Germany

**Keywords:** anxiety, depression, illness-death model, incidence, prevalence, remission

## Abstract

**Background:**

Epidemiological evidence on the incidence and remission of anxiety and depressive disorders is limited. We estimated age- and sex-specific incidence and remission rates of moderate-to-severe anxiety and depressive symptoms using the illness-death model.

**Methods:**

The German National Cohort (NAKO) is a cohort of over 200,000 participants aged 19–74 at baseline. Prevalence of probable cases, estimated with the Generalized Anxiety Disorder Scale and the Patient Health Questionnaire data 2014–2019 across five regions, was related to general mortality rates and disorder-specific mortality rate ratios in the illness-death model. The partial derivative of prevalence was modeled as a function of incidence and remission, with parameters estimated via least-squares optimization through 2,000 bootstrap resamples.

**Results:**

The highest incidence rates (per 1,000 person-years) occurred at ages 19–21 for anxiety symptoms: 4.07 (95% CI: 0.00–7.57) in women and 2.55 (0.00–4.94) in men; and at ages 28–34 for depressive symptoms: 4.41 (0.00–9.81) in women and 3.30 (0.00–7.34) in men, all in Hamburg. Remission rates (per 100 person-years) were highest at older ages. For anxiety symptoms, rates peaked at 71.8 years in women (4.10 [0.00–11.94]) and 64.2 years in men (3.00 [0.00–9.23]) in Freiburg. For depressive symptoms, the highest observed was at 74.0 years, both among women (6.61 [0.00–15.50] in Münster) and men (3.58 [0.00–11.51] in Berlin).

**Conclusions:**

Incidence and remission rates of anxiety and depressive symptoms can be estimated from prevalence and mortality data, revealing regional, sex-, and age-related variation. Validation with longitudinal data is warranted.

## Background

Anxiety disorders and depressive disorders are the most common mental disorders. Globally, 301 million and 280 million people were estimated to suffer from the respective mental disorders in 2019, with more women being affected [1]. Anxiety, characterized by excessive and persistent fear and perceived threats [2], and depression, characterized by enduring depressed mood, diminished interest, and frequent thoughts of death [3], often co-occur [4], and together inflict a high burden on the affected individuals and society as a whole [1, 5].

The body of evidence on the epidemiology of anxiety and depressive disorders in Germany is growing. In particular, a number of prevalence studies have been conducted on both depression and anxiety. For depression, population-based representative surveys (the German National Health and Examination Survey (GHS-MHS) and the German Health Interview and Examination Survey for Adults (DEGS1-MH)) showed no increase in the 12-month prevalence of major depressive disorders at 7.4% from 1997–1999 to 2009–2012, based on structured psychiatric interviews [6]. A more recent study based on administrative claims data indicated an increase in the overall prevalence of depressive disorders from 12.5% in 2009 to 15.7% in 2017 [7]. For anxiety disorders, the 12-month prevalence was estimated to be 14.5% in the 1997–1999 GHS-MHS [8] and 15.3% in the 2009–2012 DEGS1-MH [9], suggesting a possible slight increase over time. However, these slight increases may reflect the different assessment methods.

In contrast, epidemiological evidence on the incidence of these psychiatric disorders in Germany is limited. A previous study based on a health insurer’s data [10] investigated age- and sex-specific incidence of depression in the country based on a study population restricted to children and adolescents aged 6–18 years. International studies such as the Global Burden of Disease (GBD) Study provide prevalence and incidence estimates on a broad range of diseases, including depression and anxiety, at regional and country levels. However, these estimates were derived using Bayesian meta-regression modeling where, if epidemiological data were missing or sparse for a location, estimates were based on the information from proximal regions [11].

Remission is another important aspect of the life course of anxiety and depression, yet its epidemiological evidence at the population level is scarce. Anxiety disorders tend to be more chronic when individuals experience multiple episodes and suffer from comorbid mental disorders, as remission rates decrease [12]. While remission from a depressive episode is often achieved, episode recurrence is common, though less often in community settings [13]. However, approximately 30–50% individuals in mental health care settings can have persistent depression, usually after several prior episodes [13]. Age- and sex-specific remission from anxiety disorders in the German population was previously estimated based on the prevalence and incidence estimates from the GBD Study [14, 15], but there are no estimates for depression.

**Figure 1.**
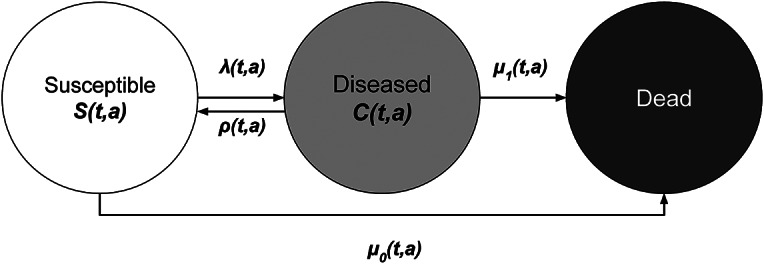
Illness-death model: In the illness-death model, the population under consideration aged *a* at time *t* is placed into three states: susceptible, diseased, and dead. As indicated by the arrows, individuals in the population move between the states at the transition rates: incidence rate 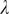 , remission rate 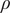 , mortality rate in the susceptible 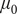 , and mortality rate in the diseased 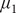 .

The illness-death model (Figure 1), a multistate modeling approach, has been shown to be useful for estimating transition rates such as incidence and remission rates, and for projecting prevalence of chronic diseases, including mental disorders [14]. One of its major strengths lies in its ability to estimate unobserved transition rates, even without direct data, by leveraging the mathematical relationship between prevalence, incidence, remission, and mortality. This feature is particularly important in mental health epidemiology, where reliable longitudinal data on remission or incidence are scarce. The application of the illness-death model can be done either in a discrete or continuous time approach [14, 16], with the former offering a more accessible entry point for public health researchers who may not be familiar with differential equations. Furthermore, age-specific incidence rates can be estimated from a series of cross-sectional prevalence data using this model [17], which provides a powerful approach for reconstructing disease dynamics from routinely collected data, even in the absence of longitudinally collected data. Here, we hypothesized that the age-specific incidence and remission rates of anxiety and depressive disorders can be estimated from cross-sectional data from multiple time points, thereby enabling more robust projections of future disease burden.

A large, longitudinal cohort such as the German National Cohort (NAKO) provides an opportunity to facilitate such an investigation. In this study, we aimed to estimate the age- and sex-specific incidence and remission rates of moderate-to-severe anxiety and depressive symptoms in the NAKO study population, applying the illness-death model to the baseline examination data from 2014 through 2019.

## Methods

### Data

The German National Cohort (NAKO, “NAKO Gesundheitsstudie”) is a population-based cohort study in Germany with over 200,000 participants aged 19–74 at baseline [18, 19]. Participants were selected through random samples stratified by age and sex, via the mandatory residence registries in the 16 study regions across the country [19]. The initial examination took place from 2014 through 2019 at 18 study centers, consisting of physical examinations, computer-assisted interviews, and self-administered questionnaires [19].

### Measures

To estimate the age- and sex-specific incidence and remission rates of anxiety and depression, we used data on the prevalence, mortality rates, and mortality rate ratios, as detailed below.

#### Prevalence of depression and anxiety

For anxiety symptom assessment, the study participants answered the self-report Generalized Anxiety Disorder Scale (GAD-7) questionnaire [20] on touchscreens [21]. The GAD-7 for the NAKO evaluates anxiety symptoms during the past 4 weeks instead of 2 weeks in the original version [22]. For depression, participants completed the Patient Health Questionnaire (PHQ-9) [23] also on touchscreens [24].

We used the participants’ assessment based on the GAD-7 and PHQ-9 as proxies to estimate the age- and sex-specific point prevalence at a study region level. For both GAD-7 and PHQ-9, the established cut-off score of ≥10 points, indicating moderate-to-severe symptom severity, was used to classify probable cases of generalized anxiety disorder and major depressive disorder. Given the long duration of the data collection period, we treated 2014–2016 and 2017–2019 as two cross-sections. To account for differences between the age and social class distribution in the study population and the sampling frame, study center-specific correction weights were constructed and provided for each participant [25, 26]. These weights were applied when reporting sample sizes (both unweighted and weighted) and estimating prevalence. The unweighted sample size reflects the actual number of participants included in the data, while the weighted sample size accounts for the sampling design and non-response, providing estimates that are more representative of the target population. The resulting age- and sex-specific prevalence of moderate-to-severe anxiety and depressive symptoms was study center-specific. Five study centers from different areas of Germany (Augsburg, Freiburg, Münster, Berlin, and Hamburg), which had a relatively large sample size, were a priori selected to perform the estimations and assess regional differences (see Supplementary Table S1 for the full list of study centers).

#### General mortality rates

Age- and sex-specific general mortality rates of the German population were obtained from the Human Mortality Database [27], which is based on the data collected by the Federal Statistical Office and statistical offices of the German states.

#### Mortality rate ratios

Estimates of the age- and sex-specific mortality rates of people with anxiety and depression are presently not available for the German population. In Denmark, however, the age-specific mortality rate ratios of the individuals with depression and anxiety disorders compared to those without the mental disorders were estimated from the national register data from 2000 to 2018 [28]. Given the stability of relative risk measures across populations [29], we utilized the Danish mortality ratios of anxiety (ICD-10: F41) and depression (ICD-10: F32) as the surrogate mortality rate ratios for the German population.

### Statistical analysis

The illness-death model conceptualizes the course of a chronic disease into multiple states (susceptible, diseased, and dead) and transitions between these states (incidence 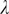 , remission 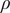 , and mortality in the susceptible 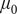 , and mortality in the diseased 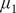 ) at the population level (Figure 1). By relating these states and transitions, the temporal change in prevalence 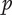 can be expressed by the following equation [30, 31]:(1)

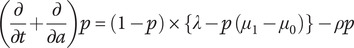

Since 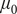 , the mortality rate among the susceptible, is often unknown, the general mortality rate 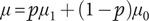 and mortality rate ratio 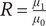 can replace 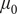 and 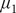 and Equation 1 becomes:(2)

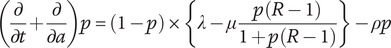


Equation 2 provides the basis for the subsequent estimation of incidence and remission rates.

We first modeled the logit of 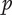 as a smooth polynomial function of time, age, sex, and study center. Then, we modeled the general mortality rates for each sex group and the mortality rate ratios for anxiety and depression, respectively. Given that the increase in prevalence is driven by the incidence (positive derivative) and that the decrease in prevalence is driven by the remission (negative derivative), we modeled the partial derivative 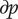 as a superposition of two Gaussian functions representing incidence 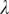 and remission 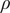 (Supplementary Figure S1), parameterized as follows:(3)

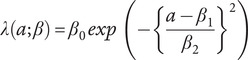


(4)

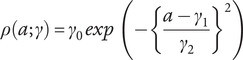

Taking this approach, the coefficients 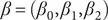 for 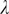 and 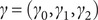 for 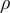 can then be found by minimizing the following sum of squares based on Equation 2, which corresponds to least-squares estimation.(5)

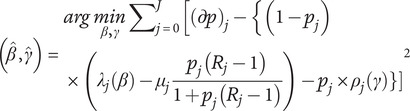

where


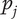
 = prevalence at age 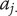



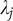
 = incidence rate at age 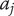



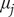
 = general mortality rate at age 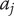



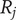
 = mortality rate ratio at age 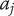



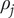
 = remission rate at age 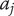


for selected ages 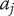 , 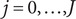 .

A polynomial with three degrees of freedom was selected based on mean squared errors from leave-one-out cross-validation. Model-fitting and parameter estimation were performed separately for anxiety and depressive symptoms. We then generated 2,000 bootstrap samples to repeat the estimation procedures and derived 95% confidence intervals (CIs) from the bootstrap replications.

### Additional analyses

To account for the diagnostic inaccuracy of self-report screening tools, we applied the published sensitivities and specificities of the PHQ-9 (78 and 87% [32]) and GAD-7 (89 and 82% [20]) through the following equation [33] to estimate age-, sex-, and study center-specific prevalence:

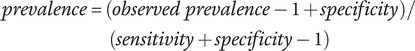



We further examined the robustness of applying mortality rate ratios from Denmark to Germany by varying 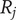 by ±15% across ages when implementing Equation 5.

### Software

All analyses were performed using R version 4.4.2 [34] and RStudio 2024.9.1.394 [35]. The analysis code is available at (https://github.com/chisato-ito/idm_nako).

## Results

### NAKO study participant characteristics

In the baseline examination data, there were a total of 204,692 participants, excluding those without information on survey weight and year of examination (Table 1 and Supplementary Figure S2). Upon applying the survey weights to correct for sampling design and non-response, the weighted sample corresponded to an estimated total population of 204,665 (Table 2). Unweighted sample size varied from 10,001 (Münster) to 31,042 (Berlin, three centers combined) with a total of 81,746 in the five selected study centers, and the weighted sample size varied from 10,002 (Münster) to 31,026 (Berlin) with a total of 81,723 (Tables 1 and 2).

**Table 1.** Characteristics of the NAKO study participants: total and by five selected study centers (unweighted)

	Overall	Select study centers				
Variable	*N* = 204,692^a^	Augsburg *N* = 20,591^a^	Freiburg *N* = 10,052^a^	Münster *N* = 10,001^a^	Berlin *N* = 31,042^a^	Hamburg *N* = 10,060^a^
**Sex**						
Men	101,416 (49.5%)	10,427 (50.6%)	5,027 (50.0%)	4,983 (49.8%)	15,293 (49.3%)	4,909 (48.8%)
Women	103,276 (50.5%)	10,164 (49.4%)	5,025 (50.0%)	5,018 (50.2%)	15,749 (50.7%)	5,151 (51.2%)
**Age**	49.9 (12.7)	50.0 (12.7)	49.9 (12.7)	49.7 (12.6)	49.8 (12.7)	49.9 (12.9)
**GAD–7**						
<10	179,062 (87.5%)	18,463 (89.7%)	8,861 (88.2%)	9,188 (91.9%)	27,364 (88.2%)	9,036 (89.8%)
≥10	9,842 (4.8%)	962 (4.7%)	403 (4.0%)	368 (3.7%)	1,556 (5.0%)	572 (5.7%)
(Missing)	15,788 (7.7%)	1,166 (5.7%)	788 (7.8%)	445 (4.4%)	2,122 (6.8%)	452 (4.5%)
**PHQ–9**						
<10	174,310 (85.2%)	18,056 (87.7%)	8,638 (85.9%)	8,989 (89.9%)	26,660 (85.9%)	8,718 (86.7%)
≥10	14,990 (7.3%)	1,437 (7.0%)	634 (6.3%)	578 (5.8%)	2,296 (7.4%)	912 (9.1%)
(Missing)	15,392 (7.5%)	1,098 (5.3%)	780 (7.8%)	434 (4.3%)	2,086 (6.7%)	430 (4.3%)

*Note*: The overall column includes all NAKO study participants in addition to the participants from the five centers selected for the analysis: Augsburg, Freiburg, Münster, Berlin, and Hamburg.

a *n* (%); mean (SD).

**Table 2.** Characteristics of the NAKO study participants: total and by five selected study centers (weighted)

	Overall	Select study centers				
Variable	*N* = 204,665^a^^,^^b^	Augsburg *N* = 20,584^a^	Freiburg *N* = 10,053^a^	Münster *N* = 10,002^a^	Berlin *N* = 31,026^a^	Hamburg *N* = 10,058^a^
**Sex**						
Men	100,707 (49.2%)	10,058 (48.9%)	4,908 (48.8%)	4,830 (48.3%)	15,224 (49.1%)	4,830 (48.0%)
Women	103,958 (50.8%)	10,526 (51.1%)	5,145 (51.2%)	5,172 (51.7%)	15,802 (50.9%)	5,228 (52.0%)
**Age**	45.7 (14.1)	46.8 (13.9)	44.4 (14.4)	42.3 (14.4)	45.2 (13.8)	44.5 (13.9)
**GAD–7**						
<10	167,827 (82.0%)	18,072 (87.8%)	8,053 (80.1%)	8,668 (86.7%)	25,837 (83.3%)	8,637 (85.9%)
≥10	13,007 (6.4%)	1,178 (5.7%)	617 (6.1%)	525 (5.2%)	2,049 (6.6%)	794 (7.9%)
(Missing)	23,832 (11.6%)	1,333 (6.5%)	1,383 (13.8%)	808 (8.1%)	3,140 (10.1%)	626 (6.2%)
**PHQ–9**						
<10	160,387 (78.4%)	17,382 (84.4%)	7,753 (77.1%)	8,359 (83.6%)	24,654 (79.5%)	8,137 (80.9%)
≥10	20,941 (10.2%)	1,916 (9.3%)	930 (9.3%)	848 (8.5%)	3,266 (10.5%)	1,330 (13.2%)
(Missing)	23,337 (11.4%)	1,285 (6.2%)	1,369 (13.6%)	795 (7.9%)	3,106 (10.0%)	591 (5.9%)

*Note:* The overall column includes all NAKO study participants in addition to the participants from the five centers selected for the analysis: Augsburg, Freiburg, Münster, Berlin, and Hamburg.

a *n* (%); mean (SD).

b Unweighted *N* = 204,692.

The weighted mean age of the participants in these study centers ranged from 42.3 (Münster) to 46.8 (Augsburg) years, and there was a slightly higher weighted proportion of women at all five centers, ranging from 50.9% (Berlin) to 52.0% (Hamburg) (Table 2). The weighted proportion of individuals with a GAD-7 sum score of 10 or higher was 6.4% overall and ranged from 5.2% (Münster) to 7.9% (Hamburg) (Table 2). The weighted proportion of individuals with a PHQ-9 sum score of 10 or higher was 10.2% overall and ranged from 8.5% (Münster) to 13.2% (Hamburg) (Table 2). The unweighted missingness ranged from 4.4 to 7.8% for GAD-7 and from 4.3 to 7.8% for PHQ-9 (Table 1).

### Incidence and remission rate estimates: anxiety

**Figure 2.**
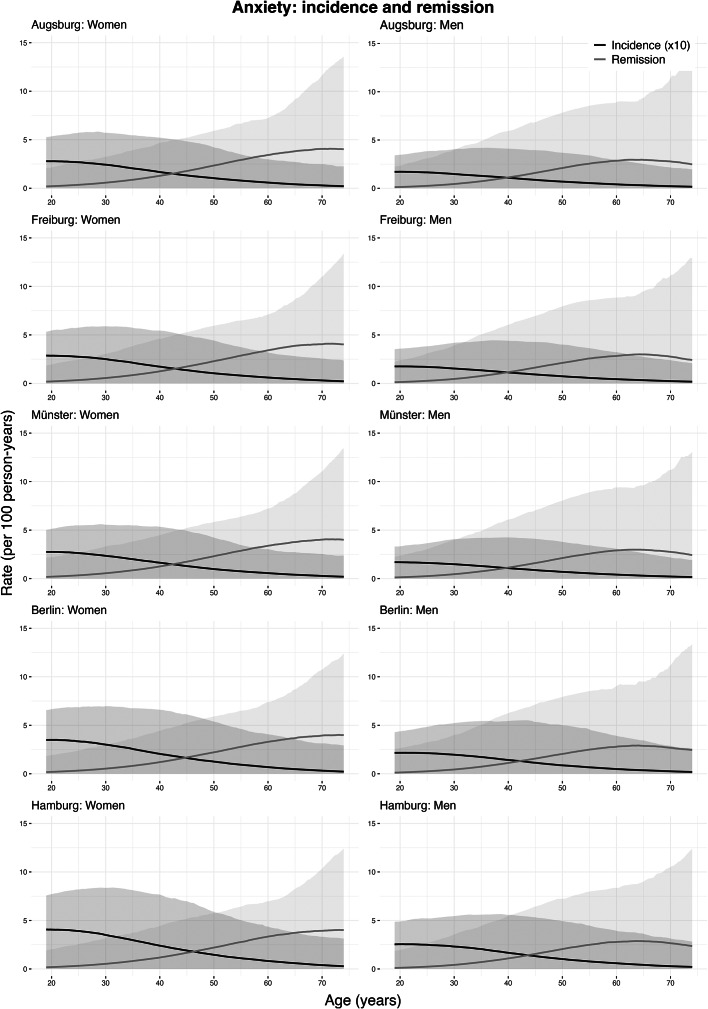
Incidence and remission rates of moderate-to-severe anxiety symptoms by age, sex, and study center (age range: 19–74 years). Shaded areas represent 95% confidence intervals, obtained by resampling. Incidence rates shown are per 1,000 person-years at risk, and remission rates are per 100 person-years at risk. The study centers are Augsburg, Freiburg, Münster, Berlin, and Hamburg. Note: Negative estimates were truncated to zero.

Figure 2 shows the estimated age-specific incidence and remission rates for moderate-to-severe anxiety symptoms by sex and study center. Incidence rates were highest at ages 19–21 in both women and men, then gradually declined with age. The highest rates were 4.07 cases/1,000 person-years (bootstrapped 95% confidence interval (CI): 0.00–7.57) in women and 2.55 (95% CI: 0.00–4.94) in men in Hamburg. Remission rates were lowest at younger ages, peaking later, with the highest observed at 71.8 years in women at 4.10 cases/100 person-years (95% CI: 0.00–11.94) and 64.2 years in men at 3.00 (95% CI: 0.00–9.23), both in Freiburg (Table 3).

**Table 3.** Peak incidence and remission rates of moderate-to-severe anxiety symptoms by sex for the five NAKO study center regions

Women				Men			
Age	Max. incidence[95% CI]^a^	Age	Max. remission[95% CI]^b^	Age	Max. incidence[95% CI]^a^	Age	Max. remission [95% CI]^b^
**Augsburg**							
19.0	2.79 [0.00, 5.26]	71.5	4.07 [0.00, 12.48]	20.0	1.71 [0.00, 3.46]	64.8	2.96 [0.00, 9.35]
**Freiburg**							
19.0	2.87 [0.00, 5.32]	71.8	4.10 [0.00, 11.94]	19.8	1.76 [0.00, 3.57]	64.2	3.00 [0.00, 9.23]
**Münster**							
20.0	2.76 [0.00, 5.14]	72.0	4.05 [0.00, 12.23]	19.0	1.69 [0.00, 3.30]	63.2	2.99 [0.00, 9.34]
**Berlin**							
20.0	3.50 [0.00, 6.66]	73.0	4.01 [0.00, 11.81]	21.0	2.17 [0.00, 4.45]	64.0	2.92 [0.00, 9.46]
**Hamburg**							
19.0	4.07 [0.00, 7.57]	73.0	4.03 [0.00, 11.87]	20.5	2.55 [0.00, 4.94]	64.0	2.88 [0.00, 8.79]

a Rate per 1,000 person-years.

b Rate per 100 person-years.

#### Regional differences

At age 20, among women, the highest incidence was 4.06 cases/1,000-person years (95% CI: 0.00–7.71) in Hamburg, while the lowest was 2.76 (95% CI: 0.00–5.14) in Münster. At age 70, the highest incidence rate was 0.40 (95% CI: 0.00–3.32) in Hamburg, and the lowest was 0.28 (95% CI: 0.00–2.54) in Münster. Among men, the highest incidence rate at age 20 was 2.55 (95% CI: 0.00–4.89) in Hamburg, and the lowest was 1.69 (95% CI: 0.00–3.33) in Münster. At age 70, the incidence rate was estimated to be highest at 0.29 (95% CI: 0.00–3.13) in Hamburg, and the lowest in Augsburg at 0.21 (95% CI: 0.00–2.15) (Table 5). For remission, among women, the highest rate at age 20 was 0.21 cases/100 person-years (95% CI: 0.00–2.14) in Augsburg, and the lowest was 0.19 (95% CI: 0.00–2.03) in Hamburg. At age 70, the highest rate was 4.05 (95% CI: 0.00–11.64) in Augsburg, and the lowest was in Hamburg at 3.93 (95% CI: 0.00–10.34). In men, at age 20, it was estimated to be highest in Berlin at 0.14 (95% CI: 0.00–2.65) and the lowest in Hamburg at 0.12 (95% CI: 0.00–1.97). At age 70, the remission rate was the highest in Augsburg at 2.78 (95% CI: 0.00–11.42), and the lowest in Hamburg at 2.65 (95% CI: 0.00–10.51) (Table 5).

### Incidence and remission rate estimates: depression

**Figure 3.**
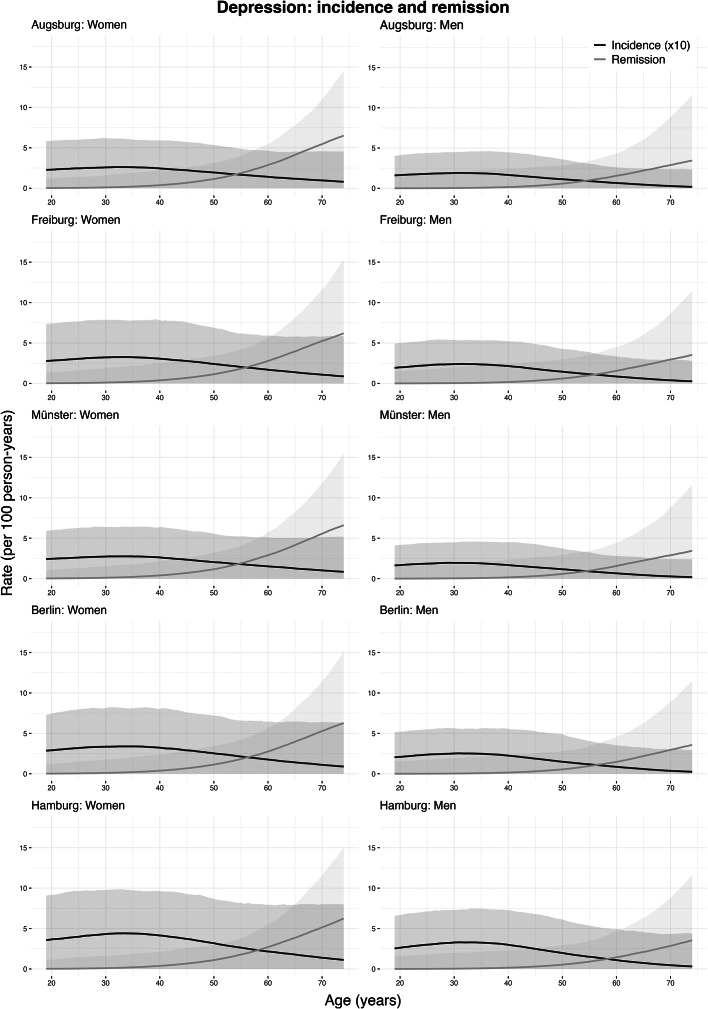
Incidence and remission rates of moderate-to-severe depressive symptoms by age, sex, and study center (age range: 19–74 years). Shaded areas represent 95% confidence intervals, obtained by resampling. Incidence rates shown are per 1,000 person-years at risk, and remission rates are per 100 person-years at risk. The study centers are Augsburg, Freiburg, Münster, Berlin, and Hamburg. Note: Negative estimates were truncated to zero.

Figure 3 shows the estimated age-specific incidence and remission rates for moderate-to-severe depressive symptoms by sex and study center. Incidence rates were highest at ages 28–34, followed by a gradual decline with age, with the peak observed at 4.41 cases/1,000 person-years (95% CI: 0.00–9.81) in women and 3.30 (95% CI: 0.00–7.34) in men in Hamburg. Remission rates were lowest at younger ages and peaked at later ages similarly across all five centers, reaching their highest observable value at age 74.0, although the true peak may be at older ages beyond the study population’s range. Specifically, remission rates reached 6.61 cases/100 person-years (95% CI: 0.00–15.50) in women in Münster and 3.58 (95% CI: 0.00–11.51) in men in Berlin (Table 4).

**Table 4.** Peak incidence and remission rates of moderate-to-severe depressive symptoms by sex for the five NAKO study center regions

Women				Men			
Age	Max. incidence[95% CI]^a^	Age	Max. remission[95% CI]^b^	Age	Max. incidence[95% CI]^a^	Age	Max. remission [95% CI]^b^
**Augsburg**							
32.0	2.63 [0.00, 6.15]	74.0	6.51 [0.00, 14.53]	30.5	1.90 [0.00, 4.50]	74.0	3.45 [0.00, 11.69]
**Freiburg**							
33.5	3.26 [0.00, 7.81]	74.0	6.20 [0.00, 15.34]	31.5	2.41 [0.00, 5.33]	74.0	3.54 [0.00, 11.39]
**Münster**							
34.0	2.75 [0.00, 6.43]	74.0	6.61 [0.00, 15.50]	28.8	1.96 [0.00, 4.48]	74.0	3.43 [0.00, 11.62]
**Berlin**							
34.5	3.39 [0.00, 8.08]	74.0	6.28 [0.00, 15.21]	31.0	2.53 [0.00, 5.58]	74.0	3.58 [0.00, 11.51]
**Hamburg**							
33.5	4.41 [0.00, 9.81]	74.0	6.23 [0.00, 15.11]	31.0	3.30 [0.00, 7.34]	74.0	3.55 [0.00, 11.64]

a Rate per 1000 person-years.

b Rate per 100 person-years.

#### Regional differences

In women, at age 20, the highest incidence rate was in Hamburg at 3.66 cases/1,000 person-years (95% CI: 0.00–9.17), and the lowest was in Augsburg at 2.30 (95% CI: 0.00–5.87). At age 70, the highest incidence rate was in Hamburg at 1.39 (95% CI: 0.00–8.01) and the lowest was 0.97 (95% CI: 0.00–4.58) in Augsburg. Among men, both at age 20 and 70, the highest incidence rate was seen in Hamburg at 2.63 (95% CI: 0.00–6.68) and 0.47 (95% CI: 0.00–4.33), respectively, and the lowest incidence rate at age 20 was in Augsburg at 1.64 (95% CI: 0.00–4.11) and at age 70 in Münster at 0.27 (95% CI: 0.00–2.42), respectively (Table 6). In terms of remission, among women, at age 20, the highest rate was 0.03 cases/100 person-years (95% CI: 0.00–1.09) in Münster, while the rate was 0.02 in other four regions. At age 70, the remission rate was highest in Münster at 5.55 (95% CI: 0.00–11.77), and lowest in Hamburg at 5.15 (95% CI: 0.00–11.60). In men, at age 20, the remission rate was 0.01 across all five regions. At age 70, the highest remission rate was 2.97 (95% CI: 0.00–8.65) in Freiburg, and the lowest was 2.86 (95% CI: 0.00–8.82) in Hamburg (Table 6).

**Table 5.**
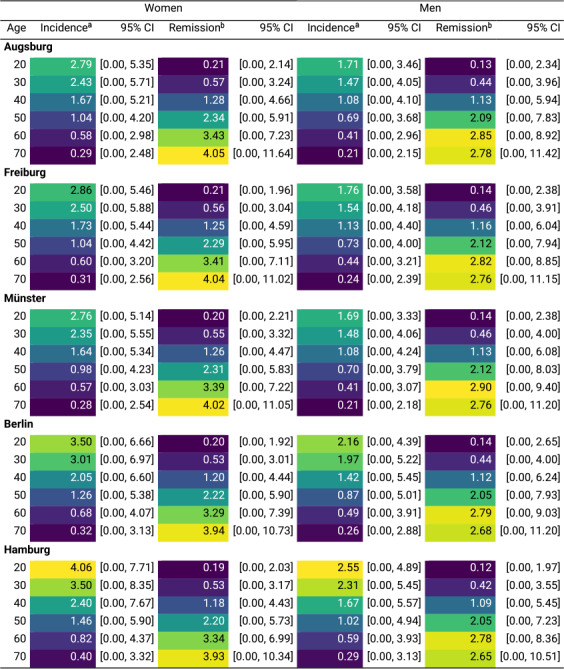
Age- and sex-specific incidence and remission rates of moderate-to-severe anxiety symptoms in the five NAKO study center regions

*Note:* Negative estimates were truncated to zero.

a Rate per 1,000 person-years.

b Rate per 100 person-years.

**Table 6.**
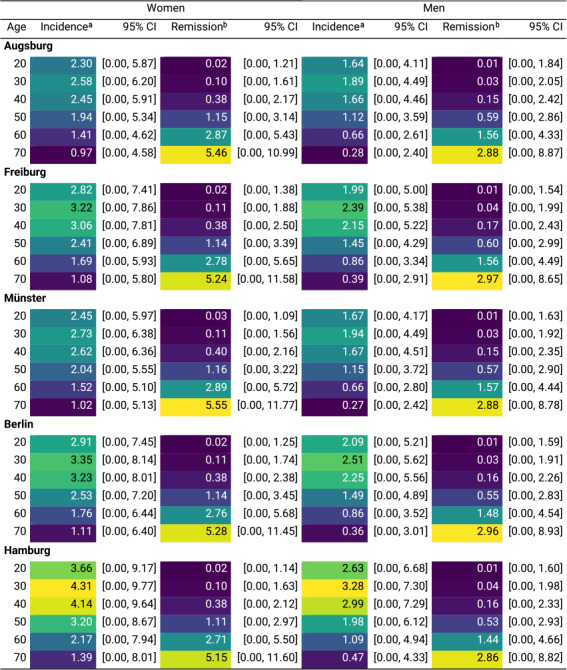
Age- and sex-specific incidence and remission rates of moderate-to-severe depressive symptoms in the five NAKO study center regions

*Note:* Negative estimates were truncated to zero.

a Rate per 1,000 person-years.

b Rate per 100 person-years.

Estimated coefficients of the partial derivative 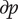 model for each study center, separately by sex and condition, are summarized in Supplementary Figures S9–S12.

### Additional analyses

Prevalence estimates adjusted for the sensitivities and specificities of the GAD-7 and PHQ-9 yielded many negative values (Supplementary Figures S4 and S6). As negative prevalence is mathematically implausible, these adjusted estimates were not used in subsequent analyses.

Varying 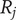 by ±15% had little impact on incidence or remission estimates. Across most ages, estimates remained largely consistent, except at older ages, where higher mortality rate ratios resulted in slightly lower remission rates and lower ratios yielded slightly higher remission rates (Supplementary Figures S13 and S14).

## Discussion

Using data from the largest German population-based cohort, we applied the illness-death model to estimate the age- and sex-specific incidence and remission rates of moderate-to-severe anxiety and depressive symptoms across different regions of Germany. By treating the NAKO baseline data from 2014 to 2019 as two sets of cross-sectional data, we modeled the prevalence as a function of time, age, sex, and study center. The resulting parsimonious prevalence model, combined with general mortality rates in Germany and mortality rate ratios for anxiety and depressive disorders expressed in functional forms, enabled us to simultaneously estimate incidence and remission rates of anxiety and depressive symptoms by leveraging the internal consistency of the illness-death model.

This approach exploits the characteristic shape of age-specific prevalence curves—where increases reflect new onset and declines reflect remission—to infer incidence and remission as a superposition of two Gaussian functions. As such, the methodology may be transferable not only to other mental disorders, but also to a wide range of chronic conditions in which remission occurs, provided that prevalence data are available across the age spectrum.

We found that incidence rates for both anxiety and depression symptoms were highest at younger ages, particularly for anxiety symptoms at ages 19–21 across sexes and regions. Remission rates peaked at younger ages in men than in women for anxiety symptoms, whereas for depressive symptoms, remission peaked similarly at age 74 or older in both sexes across the five regions. Our incidence estimates for anxiety symptoms are consistent with the existing literature, while estimates for depressive symptoms suggest a slightly later peak than previously reported. A systematic review and meta-analysis of 192 studies found that the peak age at onset for generalized anxiety disorder was 15.5 years, while the peak age at onset for depressive disorders was 19.5 years [36]. A longitudinal, register-based study in Denmark found that the age of peak incidence rates for anxiety-related disorders was 19 years in men (5.25/1,000 person-years) and 16.5 years in women (12.21/1,000 person-years) [37]. For mood disorders, the rates peaked at age 21.5 in men (3.29/1,000 person-years) and at age 20.5 in women (7.30/1,000 person-years) [37]. While our estimates are constrained by the lower age limit of 19 years in the study population, the highest values observed at and slightly above this age suggest that our model captures the common occurrence of anxiety at a young age.

This study adds a novel perspective by investigating age- and sex-specific remission rates of anxiety and depression symptoms. While previous studies have reported varying proportions of persistence and recurrence of, or remission from, anxiety and depressive disorders in community and clinical settings [12, 13, 38], long-term follow-up of a large cohort is often challenging and, thus, age-specific rates may not be attainable. It is of note that the remission rates estimated using the illness-death model approach are agnostic to treatment at the individual level. Instead, they serve as an informative measure at the population level.

### Strengths and limitations

One of the strengths of this study is the application of the illness-death model, a modern methodological approach that leverages the mathematical relationships between transition rates across health states. This approach enables the estimation of one or more transition rates, even if data on these rates are not readily available, provided that data are available on the remaining parameters [14]. In the present study, these parameters were the prevalence, general mortality rates, and mortality rate ratios of mental disorders. Another strength is the use of the data from a large, population-based cohort study. The NAKO has a large sample size of approximately 0.25% of the entire German population, with study centers spread across the country. The NAKO is planned for follow-up every 2–3 years, providing opportunities to validate our approach over time.

There are also several limitations to the present study. First, mortality rate ratios of anxiety and depressive disorders, based on ICD-10, from another country were used for the analysis, owing to the absence of such estimates in Germany. This choice is reasonable, as these estimates stem from a neighboring country of Denmark that shares many similarities with Germany, and relative risk measures are known to be stable across populations [29]. Moreover, varying 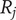 by ±15% did not result in substantial changes in the incidence or remission estimates, consistent with findings from a prior study in another disease domain [39]. Second, the NAKO weights are calculated for each study center, and not for the whole study population across Germany, due to the variation in sampling strategies [25, 26]. Consequently, it was not feasible to estimate age- and sex-specific incidence and remission rates for the entire German population. Nonetheless, the region-specific estimates and their variations offer valuable insights and may prove more effective in informing policy decisions. Given the recent development of a national mental health surveillance in Germany [40], future research should explore applying the illness-death model approach to other sources of mental disorder prevalence data that may offer broader population coverage. Third, despite some missingness in the anxiety and depression measurement (Table 1), the estimation of incidence and remission is based on prevalence derived from a complete case analysis. However, the decision not to perform multiple imputation was intentional, as the underlying mechanism of the missing data is unclear and may be missing not at random [41]. Fourth, interpretation of the mental disorder prevalence estimates based on the PHQ-9 and GAD-7 requires caution, as the use of self-report screening questionnaires rather than diagnostic interviews in estimating disease prevalence can result in overestimation [42]. Although we attempted to account for the diagnostic inaccuracy of screening tools, this approach produced many negative prevalence estimates, suggesting that the instruments’ sensitivities and specificities in the NAKO study population may differ from those reported in the literature, highlighting the need for future research. Finally, our estimation assumes that the prevalence models are well-specified and follow a functional form characterized by a gradual increase followed by a gradual decline. Such assumptions are inherent to any model-based approach. We made modeling choices as parsimonious as possible, and the resulting fits were found to be reasonable (Supplementary Figures S7 and S8).

## Conclusion

This study demonstrates the utility of the illness-death model as a life course approach to estimate age- and sex-specific incidence and remission rates of probable cases of anxiety and depressive disorders from repeated cross-sectional prevalence data. By applying this method to data from the NAKO, we identified regional differences, highlighting its applicability to large-scale, population-based mental health research. Understanding the incidence and remission rates of moderate-to-severe anxiety and depressive symptoms in specific subpopulations strengthens the epidemiological evidence for these common yet highly burdensome conditions and provides important insights for public health resource planning. As more longitudinal data become available from the NAKO and other data sources, the approach presented in this study should be further validated.

## Supporting information

10.1192/j.eurpsy.2026.10154.sm001 Ito et al. supplementary material Ito et al. supplementary material

## Data Availability

The NAKO data analyzed in this study are not publicly available due to privacy concerns. However, interested researchers can request access to the data via the NAKO transfer hub (https://transfer.nako.de/). General mortality rate data can be accessed via the Human Mortality Database (www.mortality.org). Mortality rate ratio data from Denmark are publicly available on the Danish Atlas of Disease Mortality (https://nbepi.com/atlas).

## Supplementary material

The supplementary material for this article can be found at http://doi.org/10.1192/j.eurpsy.2026.10154.

## The NAKO Investigators

Heiko Becher (Institute of Global Health, University Hospital Heidelberg, Heidelberg, Germany); Hermann Brenner (German Cancer Research Center (DKFZ), Heidelberg, Germany; Network Aging Research, Heidelberg University, Heidelberg, Germany); Stefanie Castell (Department for Epidemiology, Helmholtz Centre for Infection Research (HZI), Braunschweig, Germany); Daniela Fuhr (Department of Prevention and Evaluation, Leibniz Institute of Prevention Research and Epidemiology, Bremen, Germany; Health Sciences, University of Bremen, Bremen, Germany; Department of Health Services and Policy Research, Faculty of Public Health and Policy, London School of Hygiene and Tropical Medicine, London, UK); Hans Jörgen Grabe (Universitätsmedizin Greifswald, Greifswald, Germany); Volker Harth (Institute for Occupational and Maritime Medicine (ZfAM), University Medical Center Hamburg-Eppendorf, Hamburg, Germany); Stefan Haug (Institute of Genetic Epidemiology, Faculty of Medicine and Medical Center – University of Freiburg, Freiburg, Germany); André Karch (Institute of Epidemiology and Social Medicine, University of Münster, Münster, Germany); Thomas Keil (Institute of Social Medicine, Epidemiology and Health Economics, Charité – Universitätsmedizin Berlin, Berlin, Germany; Institute of Clinical Epidemiology and Biometry, University of Würzburg, Würzburg, Germany; State Institute of Health I, Bavarian Health and Food Safety Authority, Erlangen, Germany); Lena Koch-Gallenkamp (Division of Clinical Epidemiology and Aging Research, German Cancer Research Center (DKFZ), Heidelberg, Germany); Lilian Krist (Institute of Social Medicine, Epidemiology and Health Economics, Charité – Universitätsmedizin Berlin, Berlin, Germany); Michael Leitzmann (Institute for Epidemiology and Preventive Medicine, University of Regensburg, Regensburg, Germany); Claudia Meinke-Franze (Institute for Community Medicine, University Medicine Greifswald, Greifswald, Germany); Rafael Mikolajczyk (Institute for Medical Epidemiology, Biometrics, and Informatics (IMEBI), Interdisciplinary Center for Health Sciences, Medical Faculty of the Martin Luther University Halle-Wittenberg, Halle (Saale), Germany; German Center for Mental Health, Site Halle-Jena-Magdeburg, Halle (Saale), Germany); Katharina Nimptsch (Molecular Epidemiology Research Group, Max Delbrück Center for Molecular Medicine in the Helmholtz Association (MDC), Berlin, Germany); Nadia Obi (Institute for Occupational and Maritime Medicine (ZfAM), University Medical Center Hamburg-Eppendorf, Hamburg, Germany); Annette Peters (Institute of Epidemiology, Helmholtz Zentrum München – German Research Center for Environmental Health (GmbH), Neuherberg, Germany; Institute for Medical Information Processing, Biometry and Epidemiology, Medical Faculty, Ludwig-Maximilians-Universität München, Munich, Germany); Tobias Pischon (Molecular Epidemiology Research Group, Max-Delbrück-Center for Molecular Medicine in the Helmholtz Association (MDC), Berlin, Germany; Biobank Technology Platform, Max-Delbrück-Center for Molecular Medicine in the Helmholtz Association (MDC), Berlin, Germany; Charité - Universitätsmedizin Berlin, Berlin, Germany); Carsten Schmidt (Institute for Community Medicine, University Medicine Greifswald, Greifswald, Germany); Matthias Schulze (Department of Molecular Epidemiology, German Institute of Human Nutrition, Potsdam-Rehbruecke, Nuthetal, Germany; Institute of Nutritional Science, University of Potsdam, Nuthetal, Germany).
