# Supplementary material for: Applying the illness-death model to estimate the incidence and remission of severe anxiety and depressive symptoms in the German National Cohort (NAKO)
Source: Eur Psychiatry. 2026 Jan 29;69(1):e23. doi: 10.1192/j.eurpsy.2026.10154 (PMC12925673; doi:10.1192/j.eurpsy.2026.10154)

# Supplementary Material

**Table S1.** List of the German National Cohort (NAKO) study centers

**Figure S1.** Illustration of the partial derivative $\partial p$ model

**Figure S2.** Flow diagram of study participants included in the analysis

**Figure S3.** Prevalence of moderate-to-severe anxiety symptoms in 2014-16 and 2017-19 by age, sex, and study center

**Figure S4.** Prevalence of moderate-to-severe anxiety symptoms in 2014–16 and 2017–19 by age, sex, and study center incorporating the sensitivity and specificity of GAD-7

**Figure S5.** Prevalence of moderate-to-severe depressive symptoms in 2014–16 and 2017–19 by age, sex, and study center

**Figure S6.** Prevalence of moderate-to-severe depressive symptoms in 2014–16 and 2017–19 by age, sex, and study center incorporating the sensitivity and specificity of PHQ-9

**Figure S7.** Comparison of the observed and predicted prevalence of moderate-to-severe anxiety symptoms in the years 2014–16 and 2017–19

**Figure S8.** Comparison of the observed and predicted prevalence of moderate-to-severe depressive symptoms in the years 2014–16 and 2017–19

**Figure S9.** Estimated coefficients of the partial derivative $\partial p$ model for anxiety symptoms among women.

**Figure S10.** Estimated coefficients of the partial derivative $\partial p$ model for anxiety symptoms among men.

**Figure S11.** Estimated coefficients of the partial derivative $\partial p$ model for depressive symptoms among women.

**Figure S12.** Estimated coefficients of the partial derivative $\partial p$ model for depressive symptoms among men.

**Figure S13.** Incidence and remission rates of moderate-to-severe anxiety symptoms by age, sex, and study center under varying mortality rate ratios.

**Figure S14.** Incidence and remission rates of moderate-to-severe depressive symptoms by age, sex, and study center under varying mortality rate ratios.

**Table S1.** List of the German National Cohort (NAKO) study centers

| Study Center | State |
| --- | --- |
| **Augsburg^a^** | Bavaria |
| Regensburg | Bavaria |
| Mannheim | Baden-Württemberg |
| **Freiburg^a^** | Baden-Württemberg |
| Saarbrücken | Saarland |
| Essen | North Rhine-Westphalia |
| **Münster^a^** | North Rhine-Westphalia |
| Düsseldorf | North Rhine-Westphalia |
| Halle | Saxony-Anhalt |
| Leipzig | Saxony |
| **Berlin North^a,b^** | Berlin |
| **Central Berlin^a,b^** | Berlin |
| **Berlin South^a,b^** | Berlin |
| Hanover | Lower Saxony |
| **Hamburg^a^** | Hamburg |
| Bremen | Bremen |
| Kiel | Schleswig-Holstein |
| Neubrandenburg | Mecklenburg-Vorpommern |

**^a^**Incidence and remission rates were estimated for these study centers.

**^b^**The three study centers in Berlin were treated as one center.

**Figure S1.** Illustration of the partial derivative $\partial p$ modeled as a superposition of two Gaussian functions. The positive component, driven by disease incidence, is parameterized by $\beta=\left( \beta_{0},\beta_{1},\beta_{2} \right)$, while the negative component, corresponding to remission, is parameterized by $\gamma=\left( \gamma_{0},\gamma_{1},\gamma_{2} \right)$. The solid blue line indicates the age range with available data in the NAKO study population (age: 19–74). The dotted blue lines extend into gray-shared areas to show the functional form in the age ranges where data are absent, but no estimates are made in these regions.

**
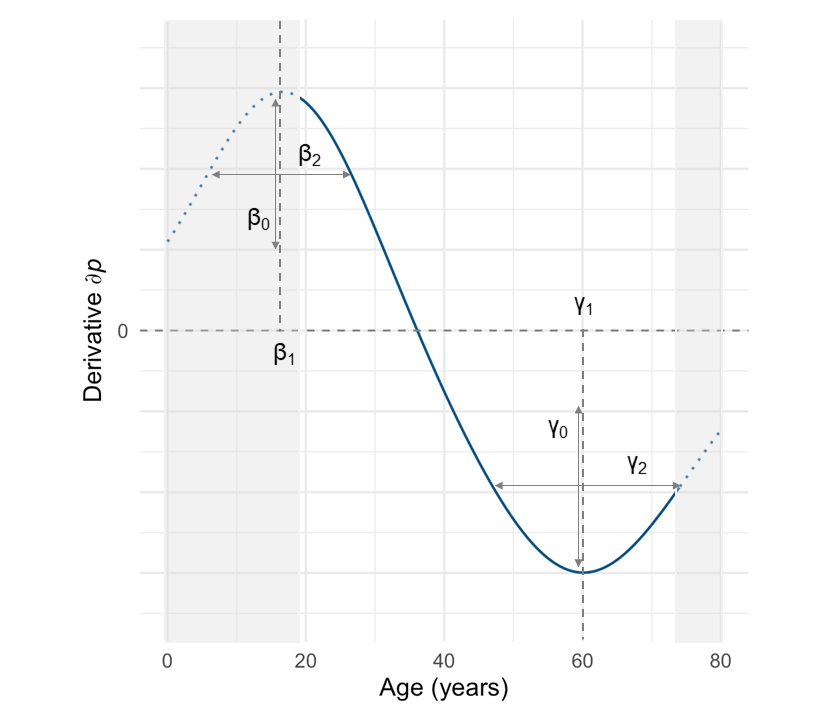
**

**Figure S2.** Flow diagram of NAKO study participants included in the analysis.


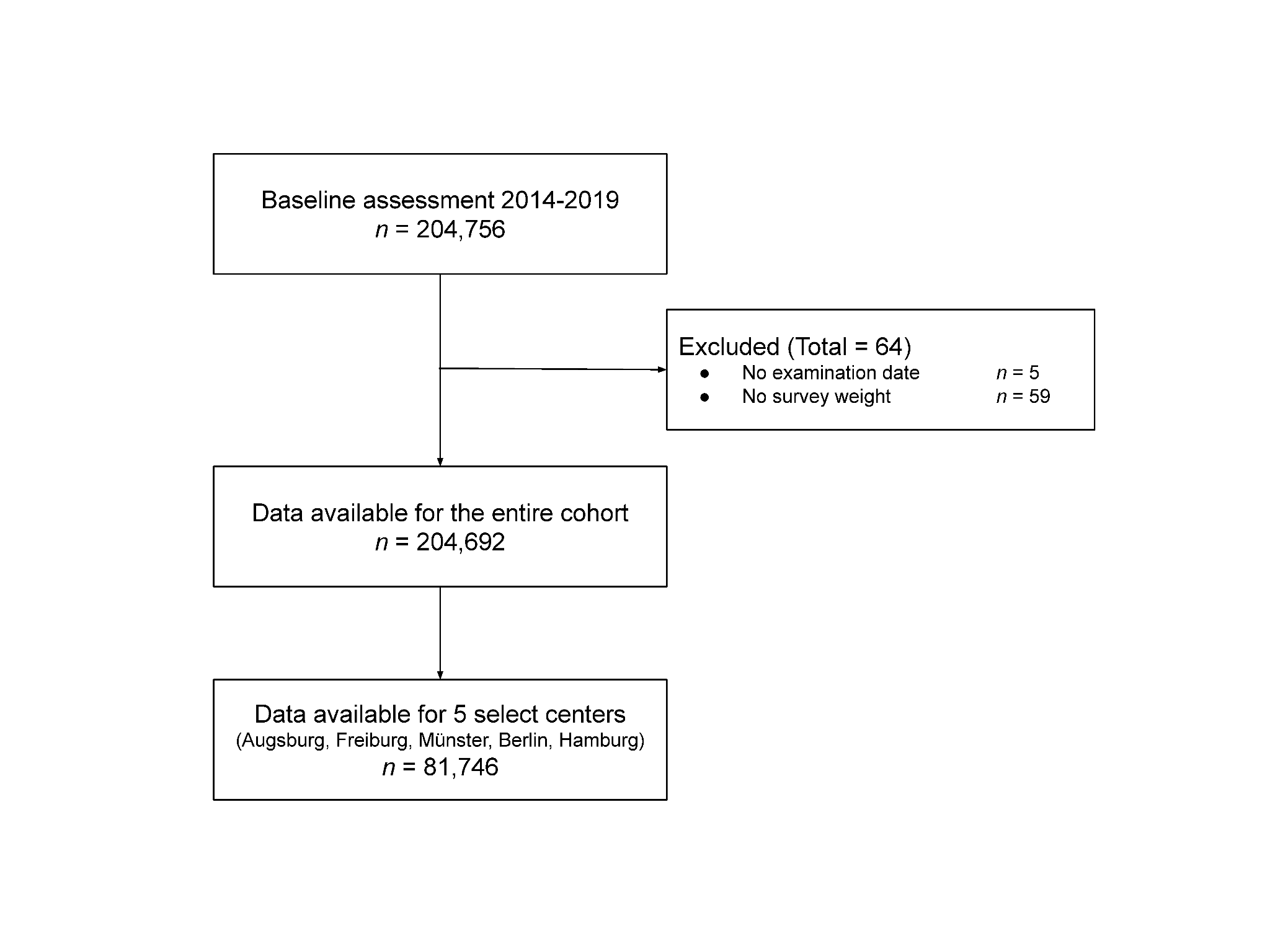


**Figure S3.** Prevalence of moderate-to-severe anxiety symptoms in 2014-16 and 2017-19 by age, sex, and study center (Augsburg, Freiburg, Münster, Berlin, and Hamburg), estimated using the 2,000 bootstrap samples. Vertical lines indicate 95% confidence intervals.


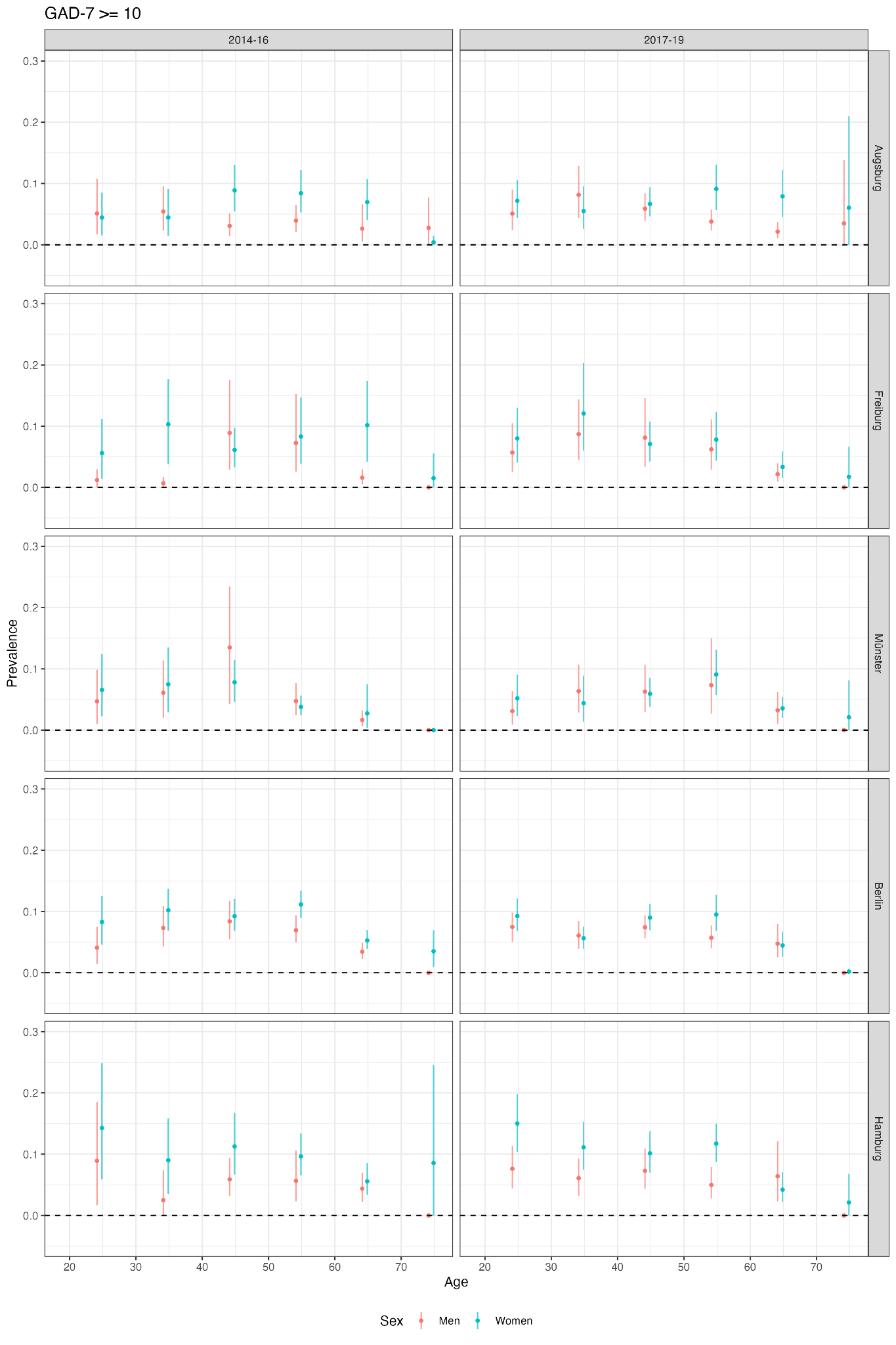


**Figure S4.** Prevalence of moderate-to-severe anxiety symptoms in 2014–16 and 2017–19 by age, sex, and study center (Augsburg, Freiburg, Münster, Berlin, and Hamburg), incorporating the sensitivity and specificity of GAD-7 and estimated using the 2,000 bootstrap samples. Vertical lines indicate 95% confidence intervals.

**
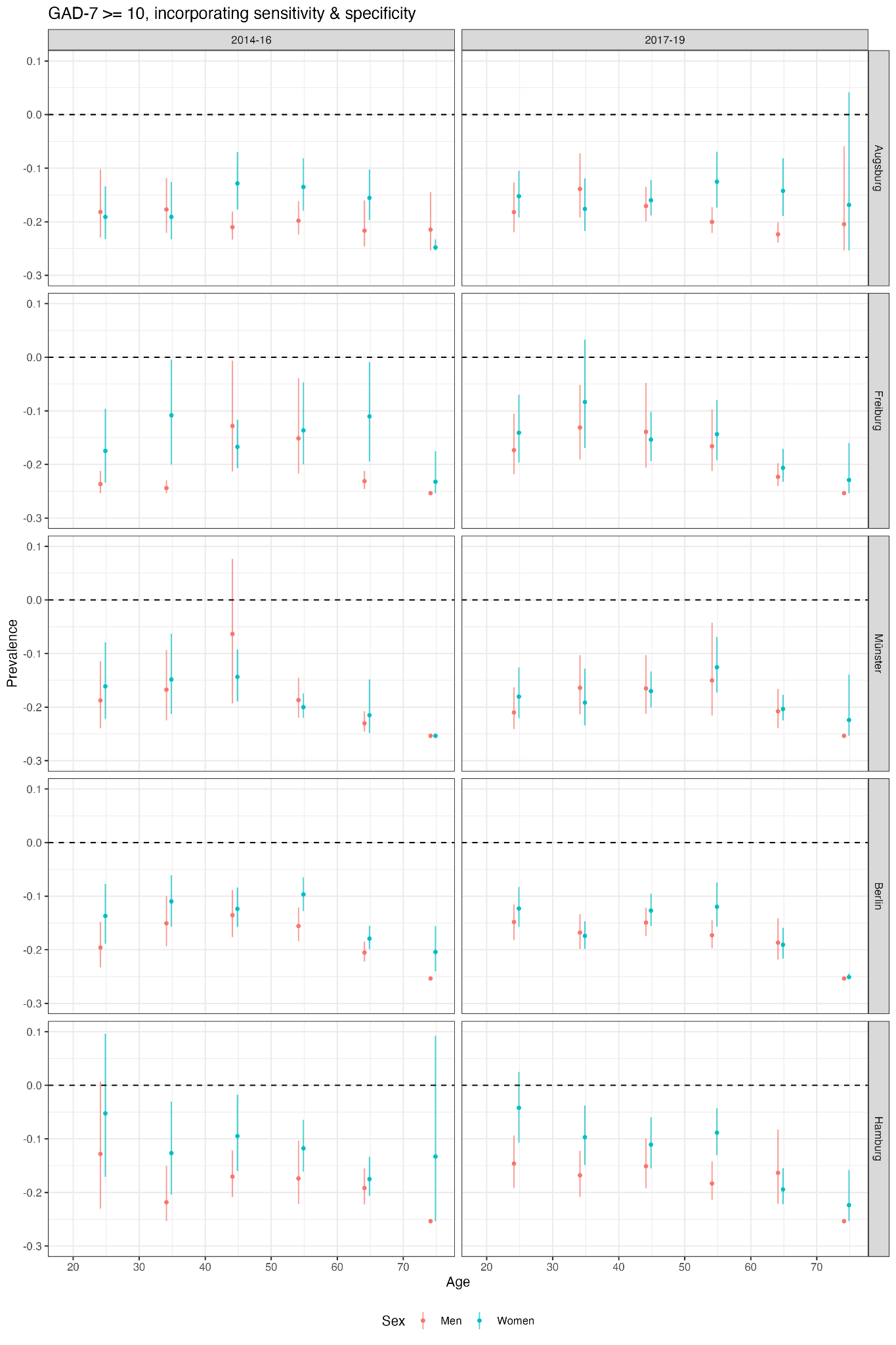
**

**Figure S5.** Prevalence of moderate-to-severe depressive symptoms in 2014–16 and 2017–19 by age, sex, and study center (Augsburg, Freiburg, Münster, Berlin, and Hamburg), estimated using the 2,000 bootstrap samples. Vertical lines indicate 95% confidence intervals.


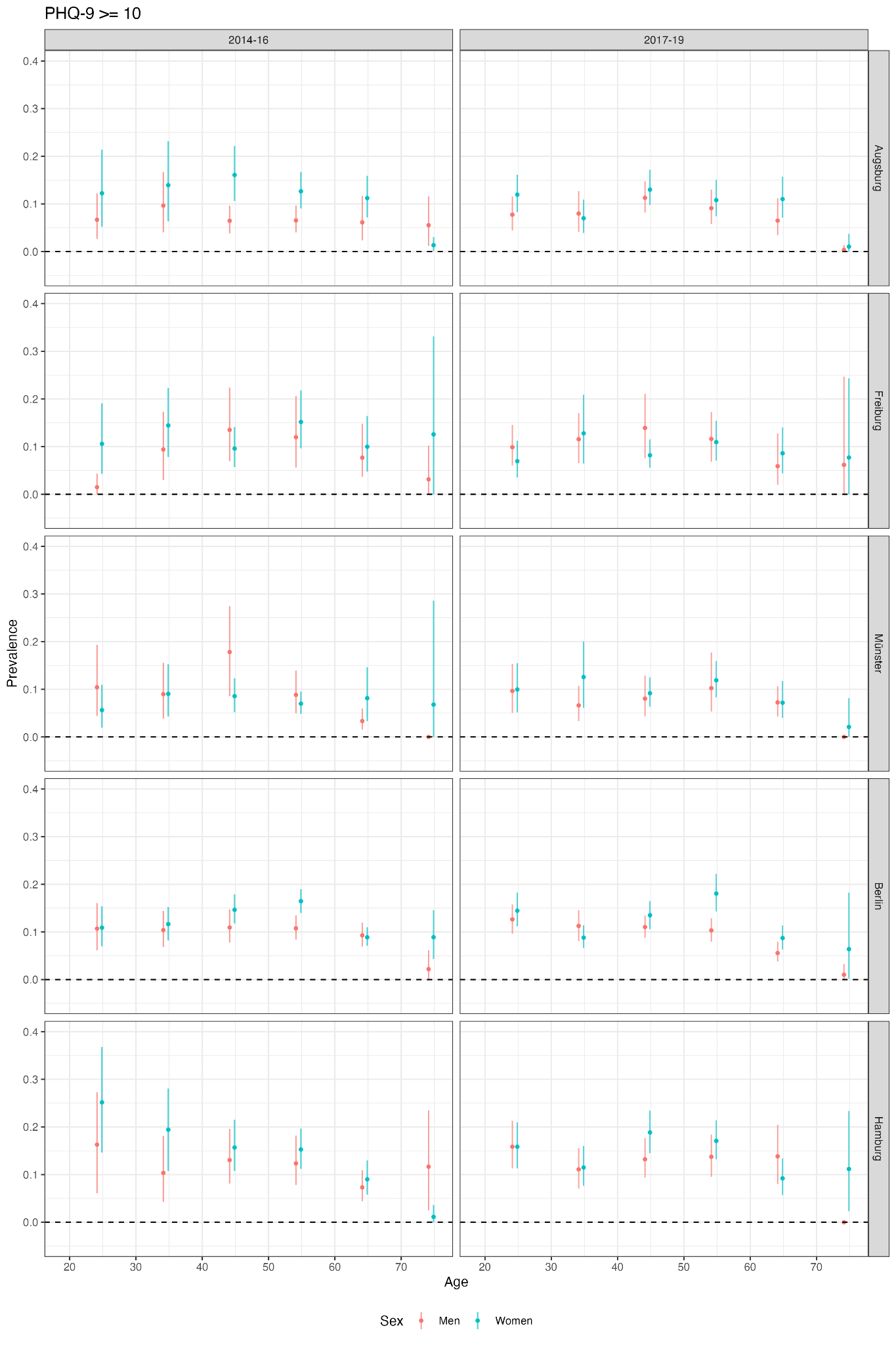


**Figure S6.** Prevalence of moderate-to-severe depressive symptoms in 2014–16 and 2017–19 by age, sex, and study center (Augsburg, Freiburg, Münster, Berlin, and Hamburg), incorporating the sensitivity and specificity of PHQ-9 and estimated using the 2,000 bootstrap samples. Vertical lines indicate 95% confidence intervals.


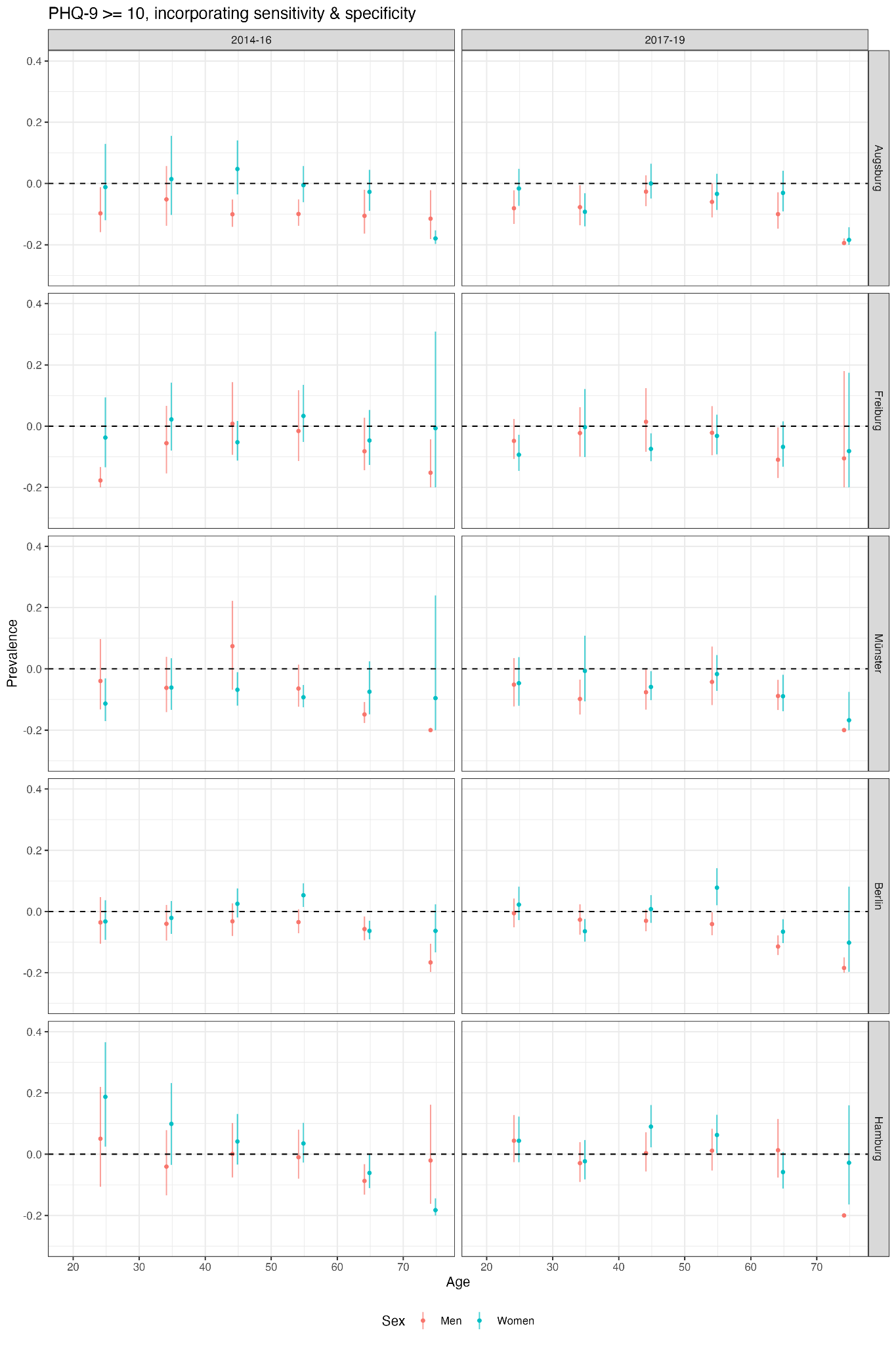


**Figure S7.** Comparison of the observed and predicted prevalence of moderate-to-severe anxiety symptoms in the years 2014–16 and 2017–19 at five study centers (Augsburg, Freiburg, Münster, Berlin, and Hamburg). The observed values are indicated by “x,” with 95% confidence intervals, shown as vertical lines. The predicted values from the prevalence model are shown as dotted lines.


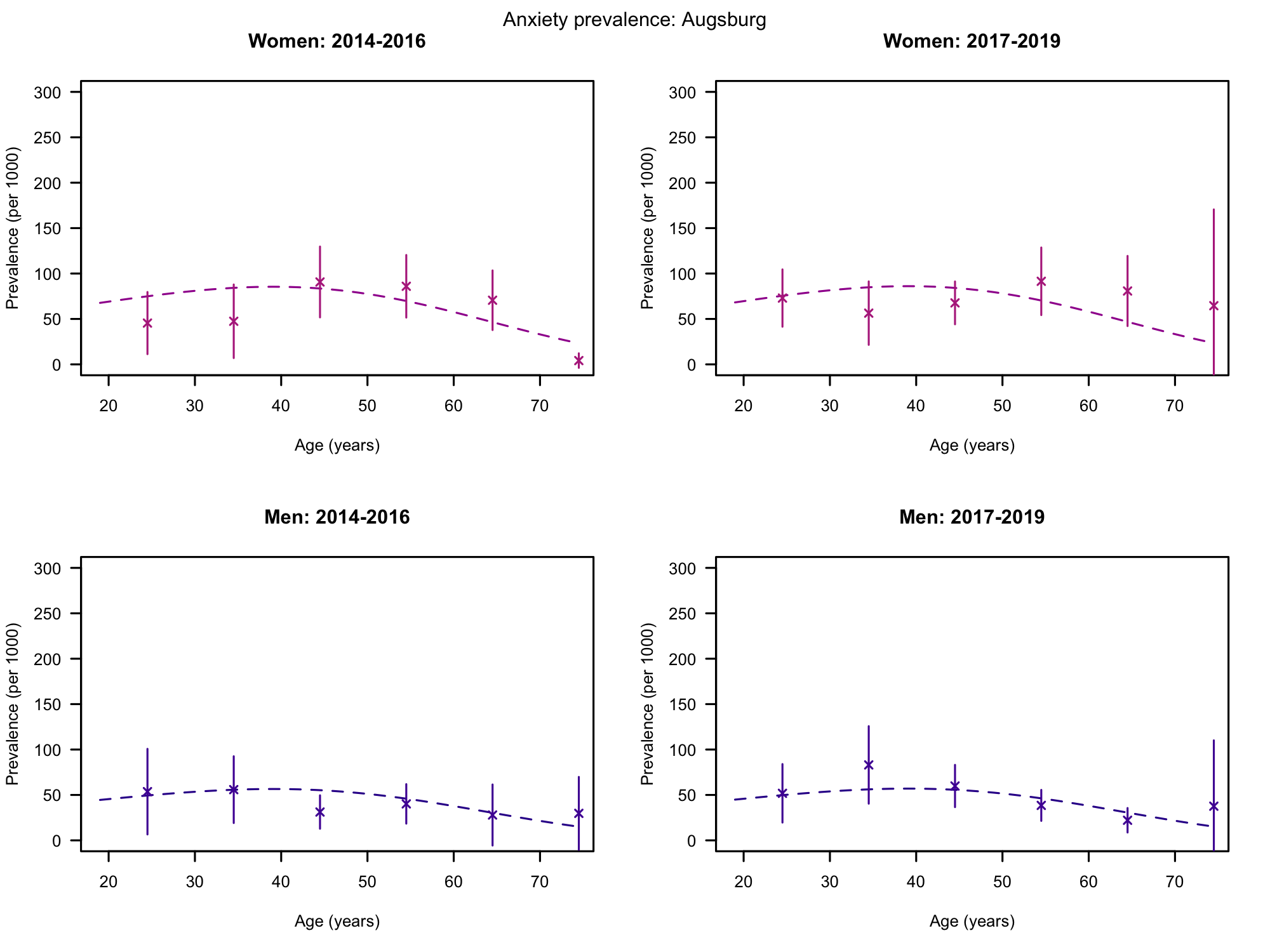

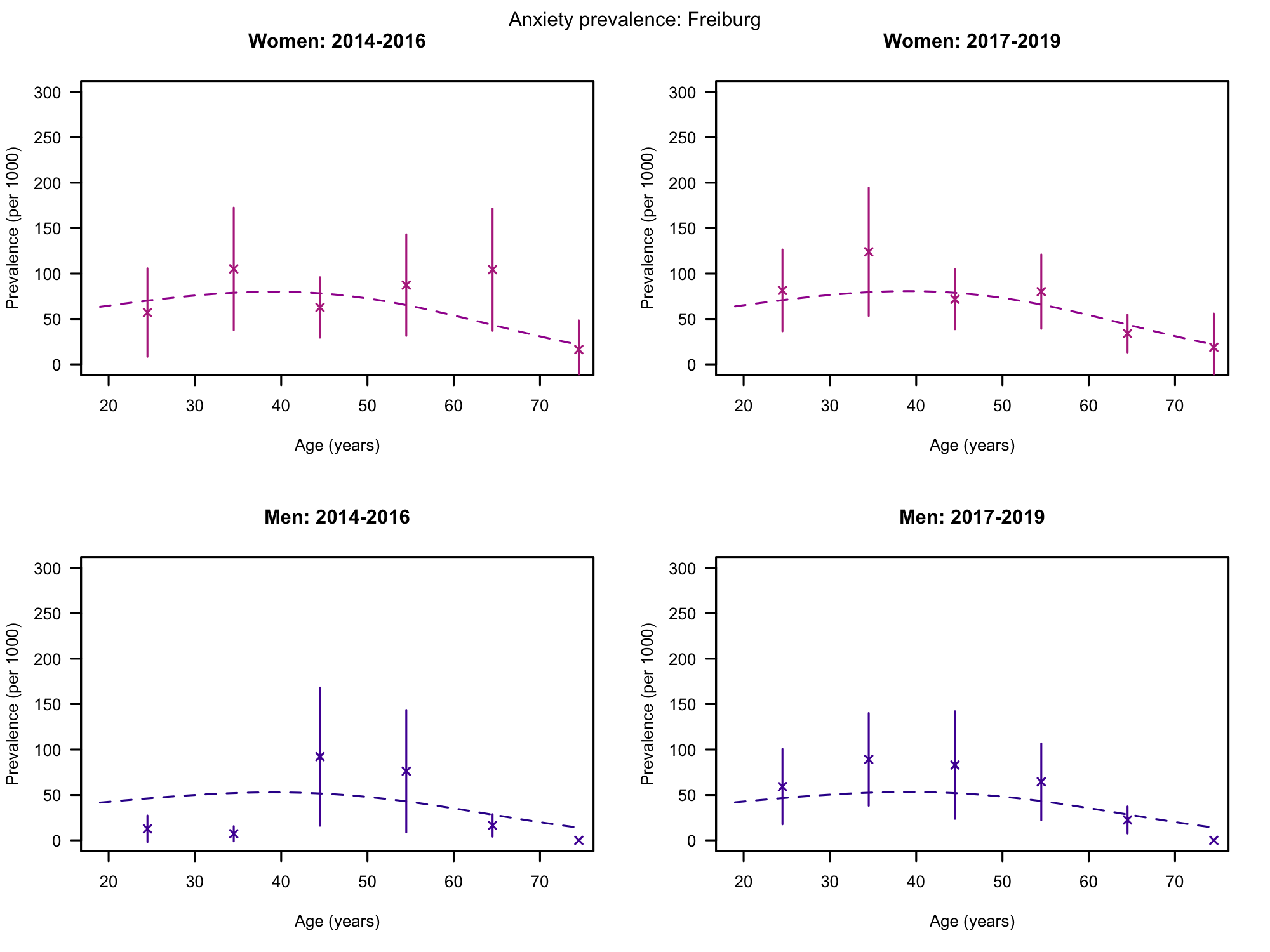

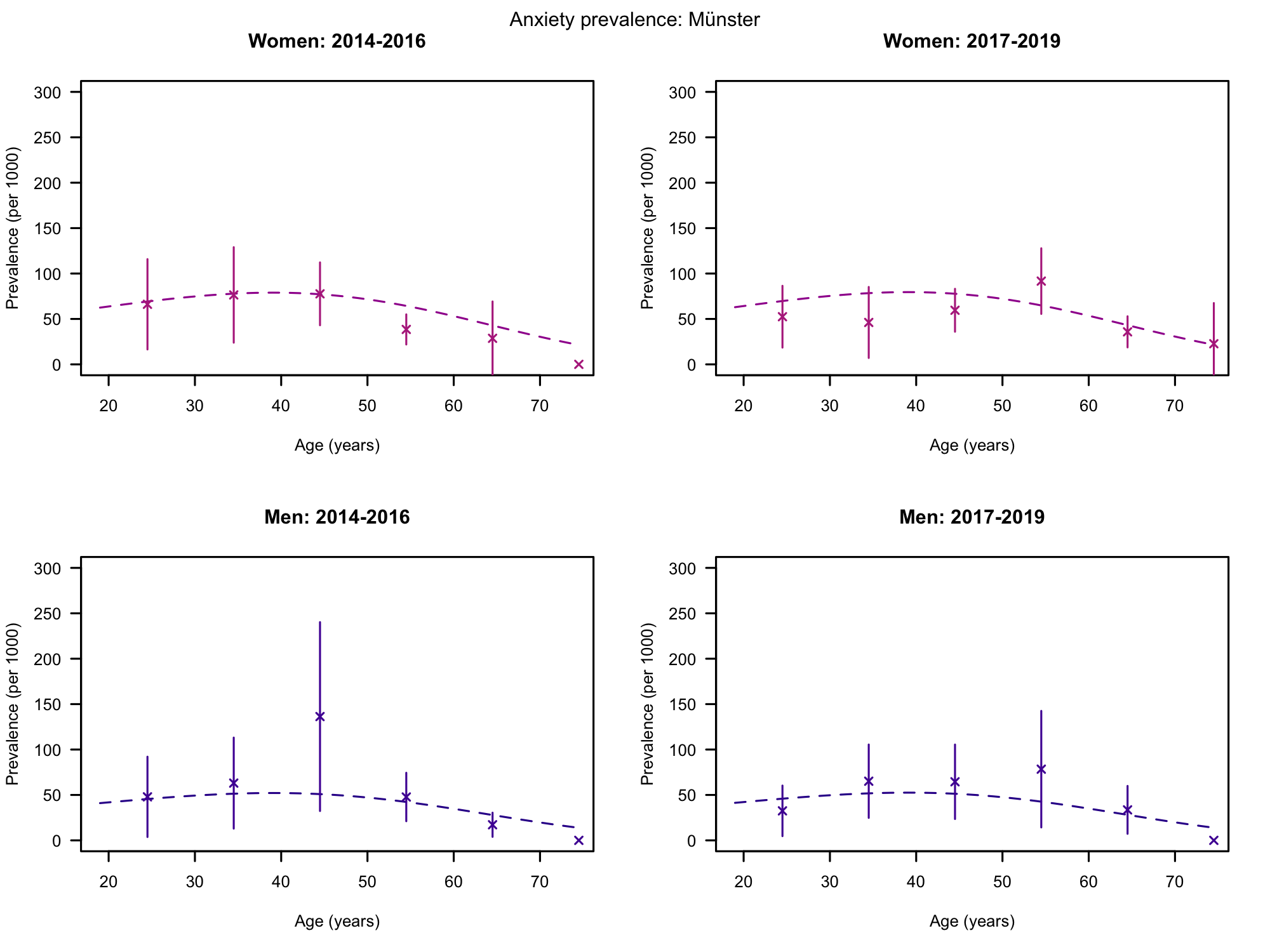

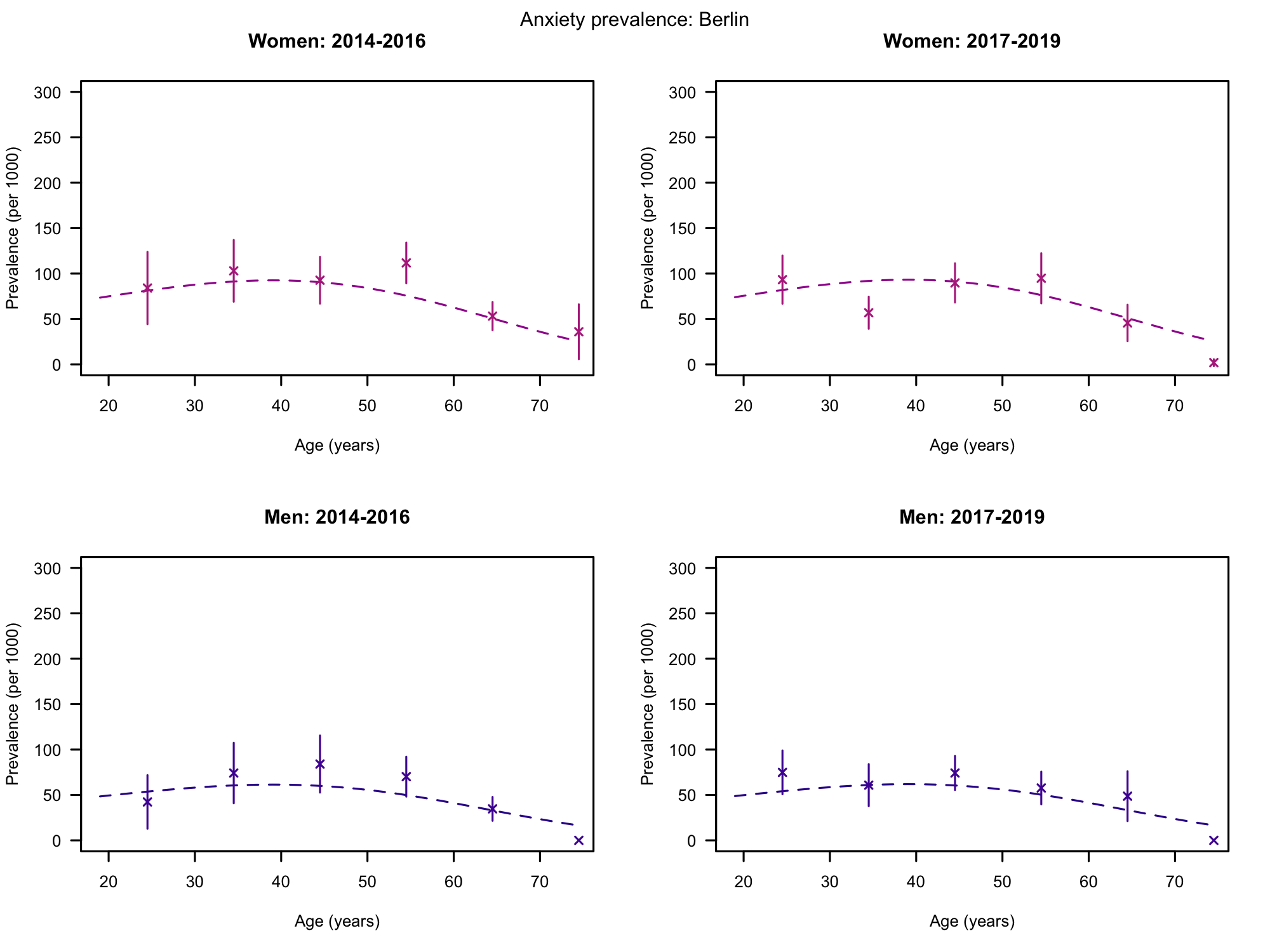

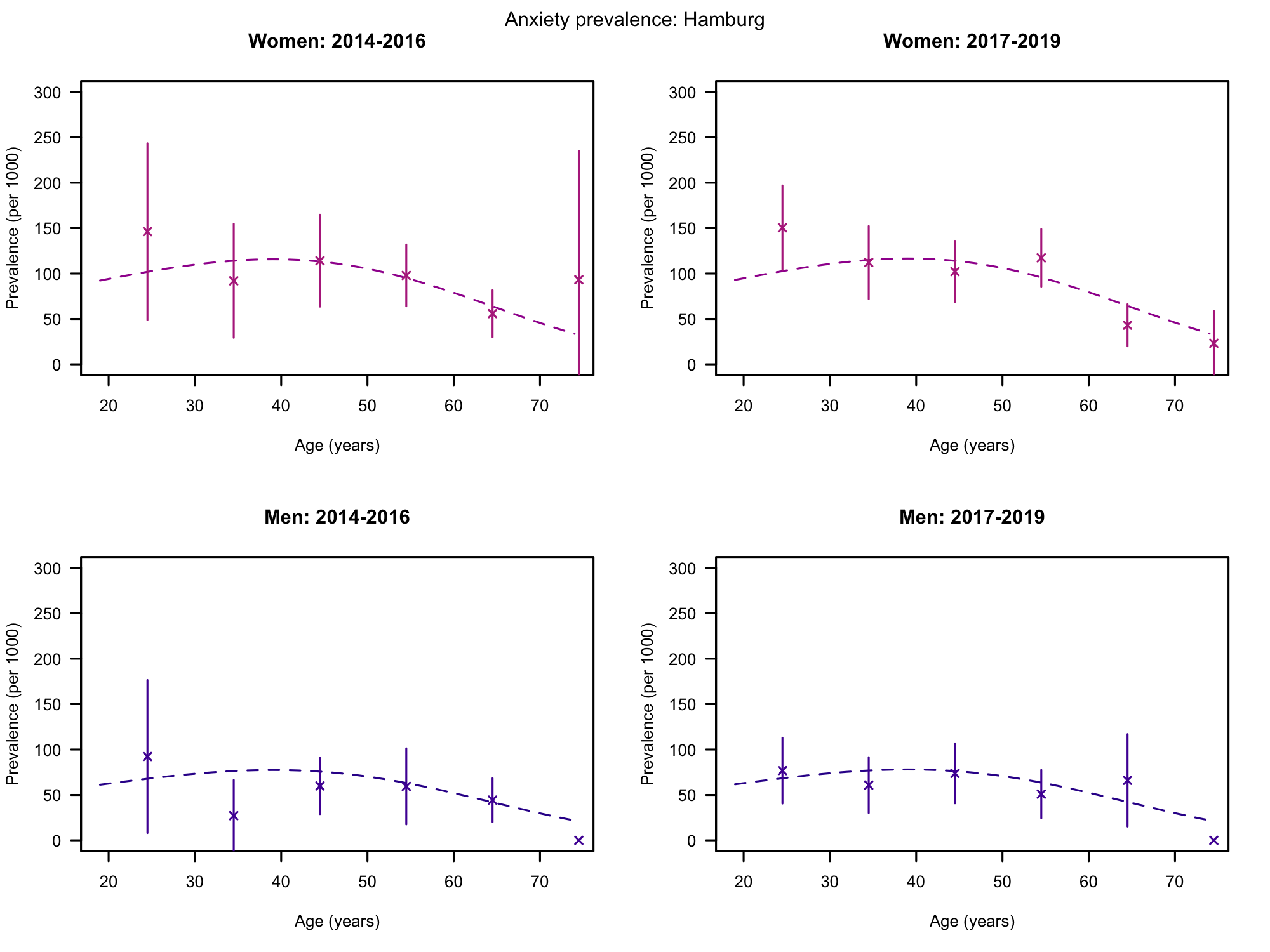


**Figure S8.** Comparison of the observed and predicted prevalence of moderate-to-severe depressive symptoms in the years 2014–16 and 2017–19 at five study centers (Augsburg, Freiburg, Münster, Berlin, and Hamburg). The observed values are indicated by “x,” with 95% confidence intervals, shown as vertical lines. The predicted values from the prevalence model are shown as dotted lines.


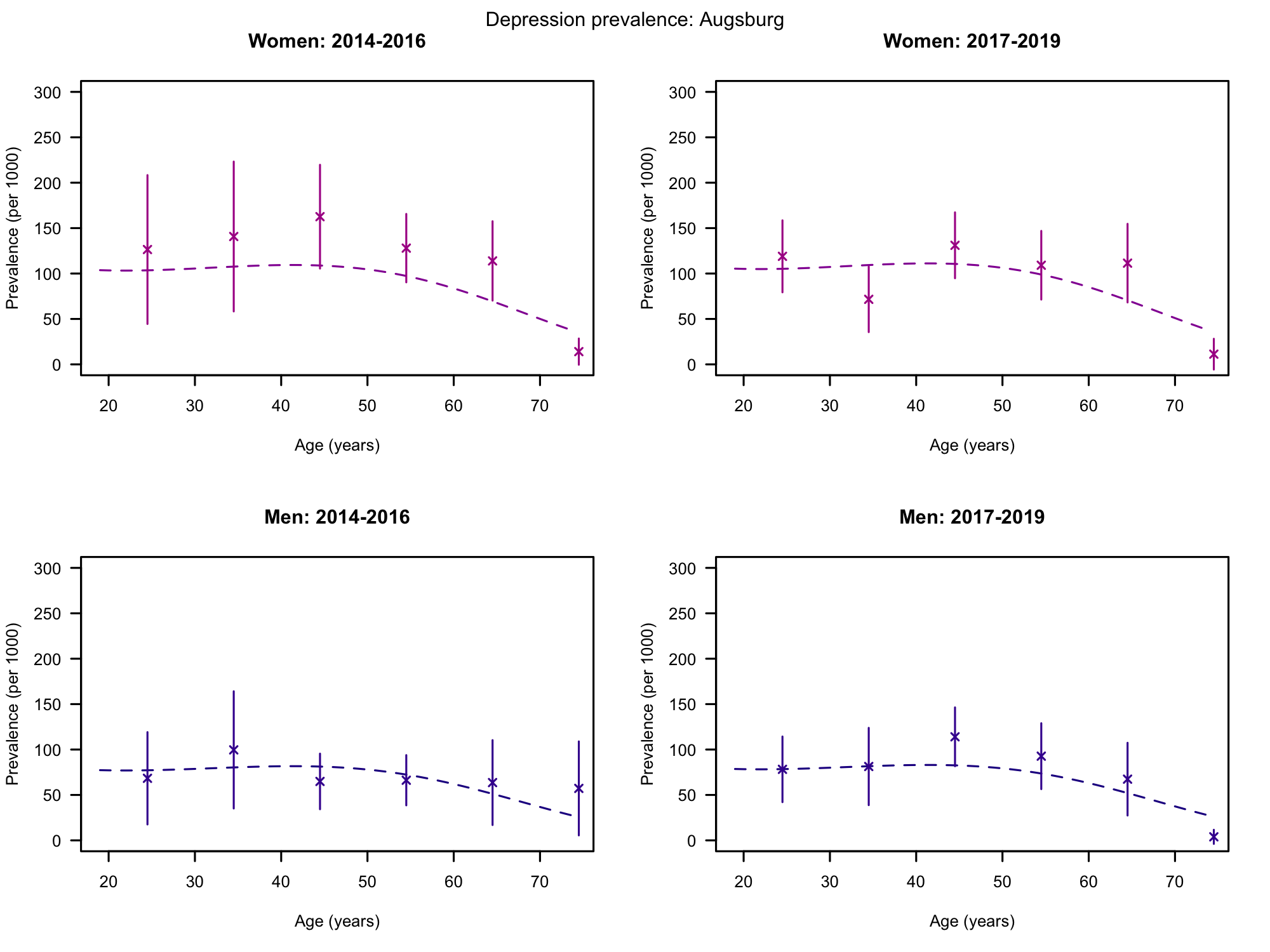

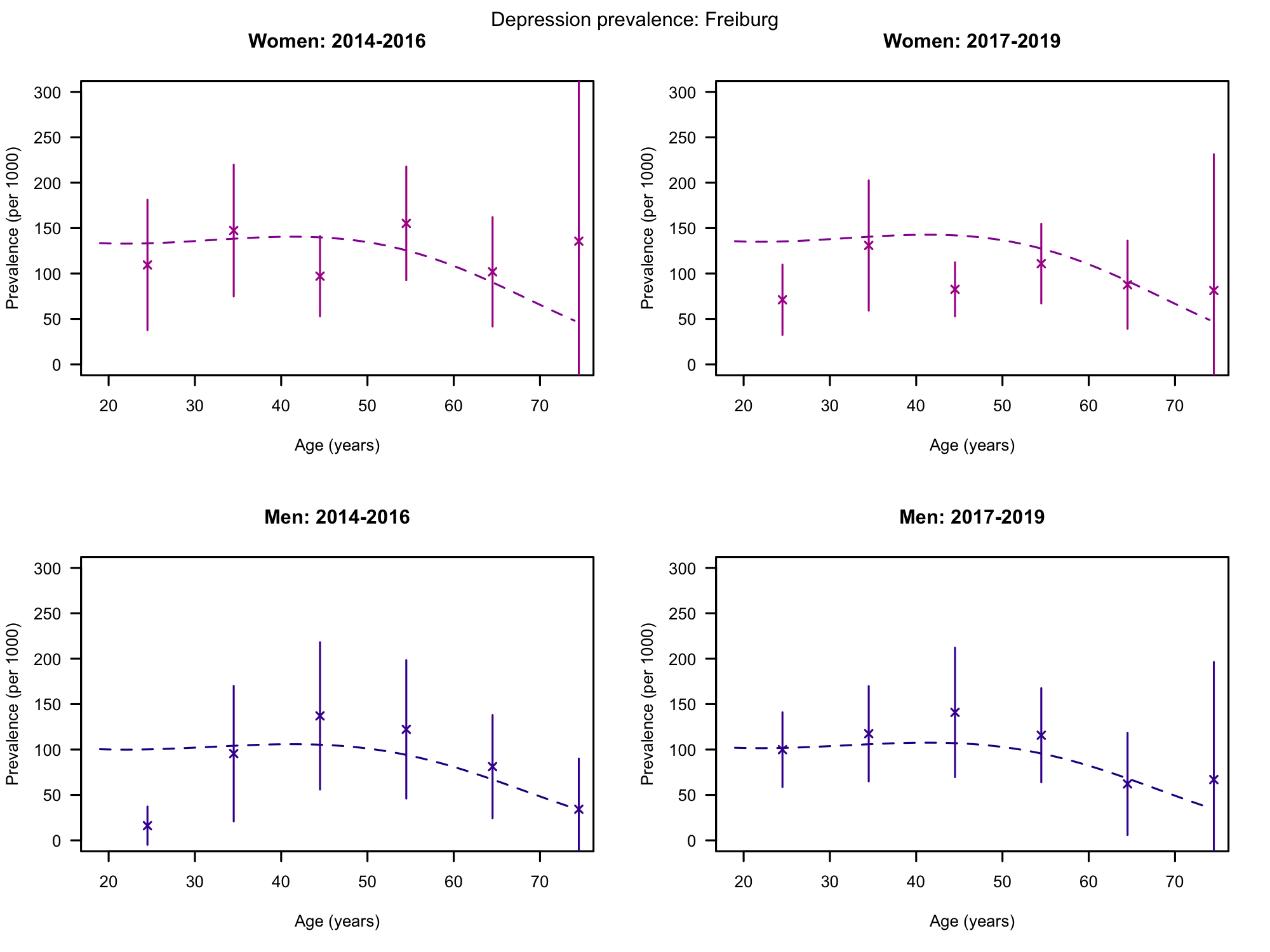

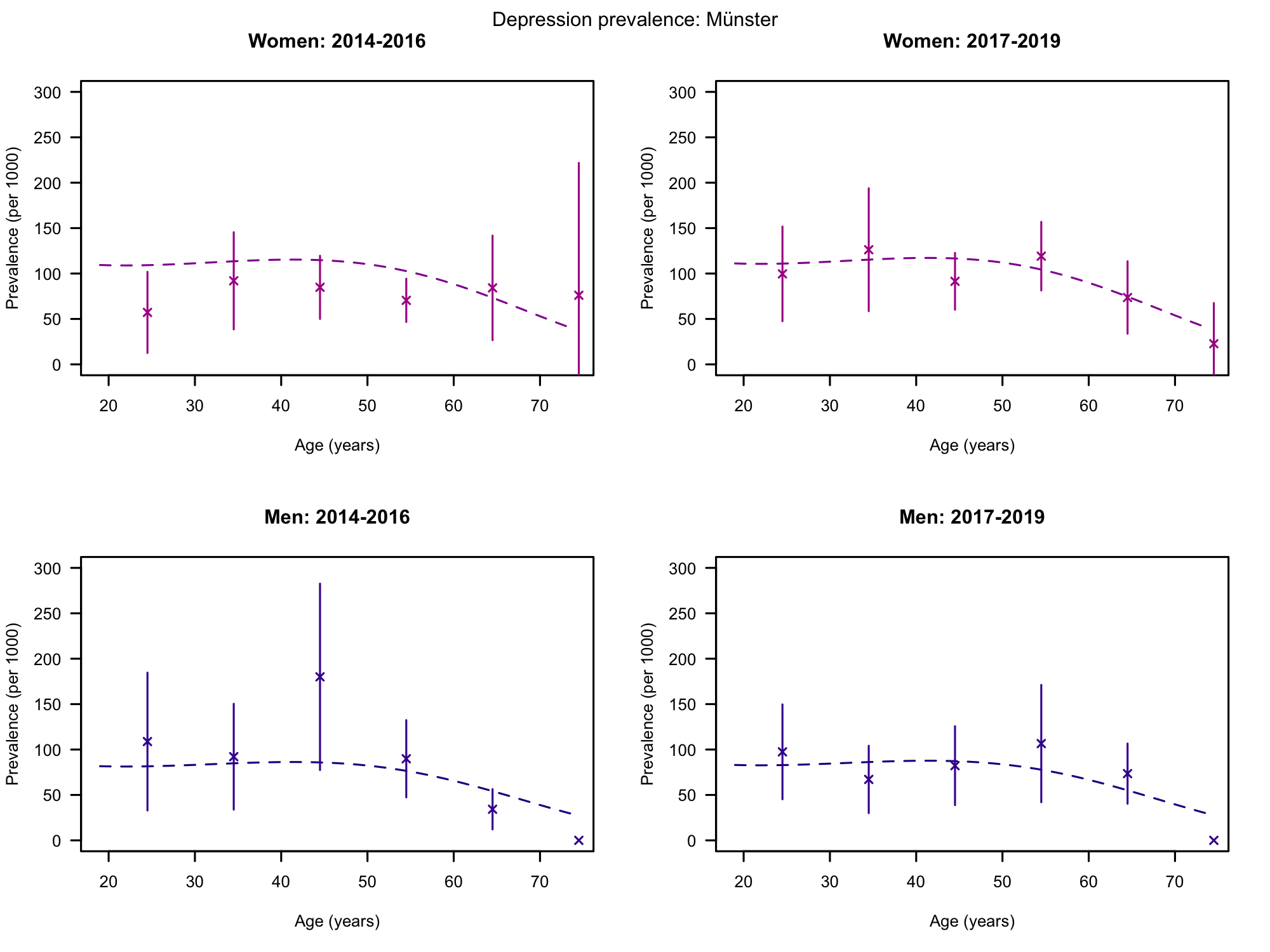

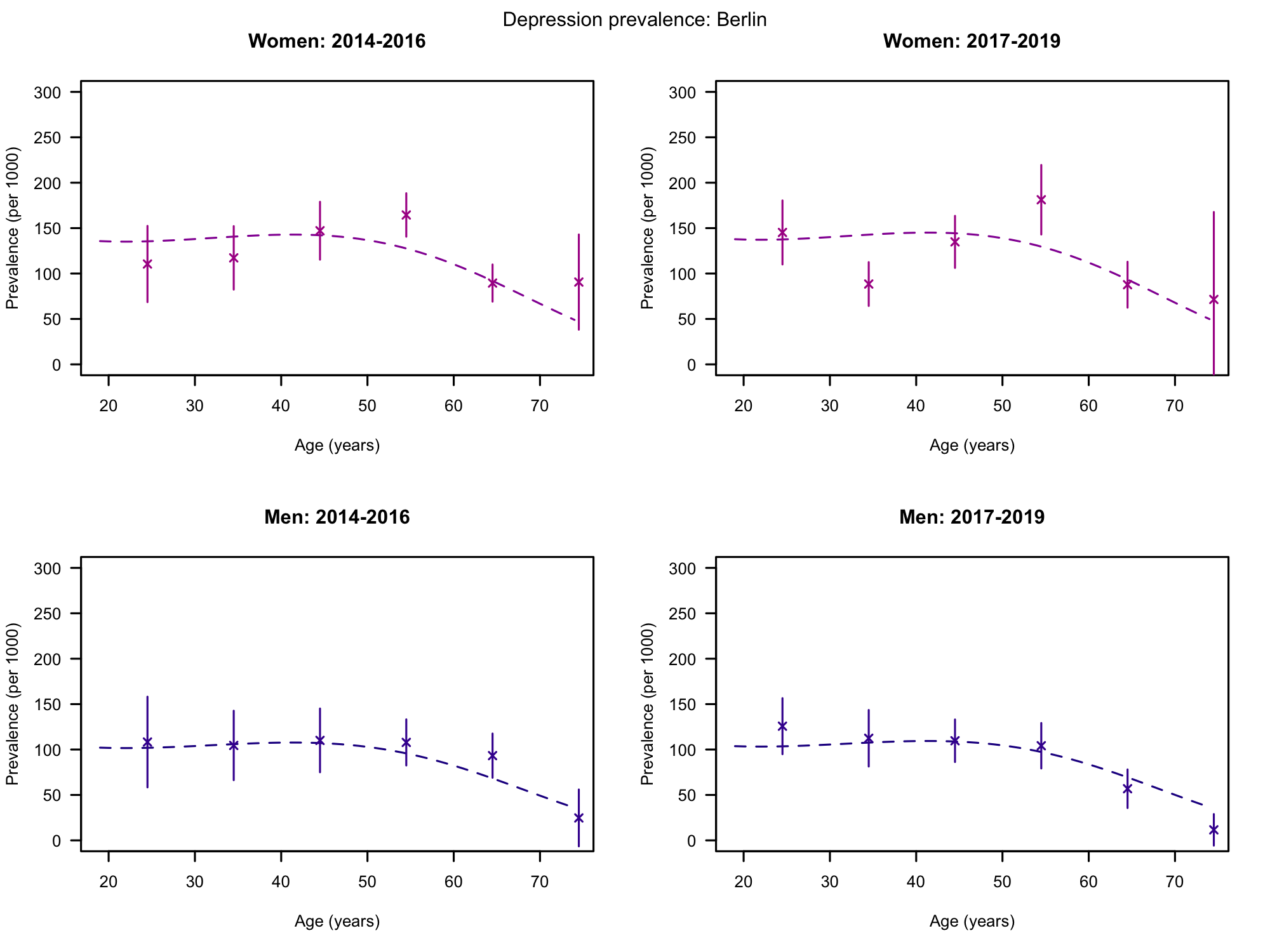

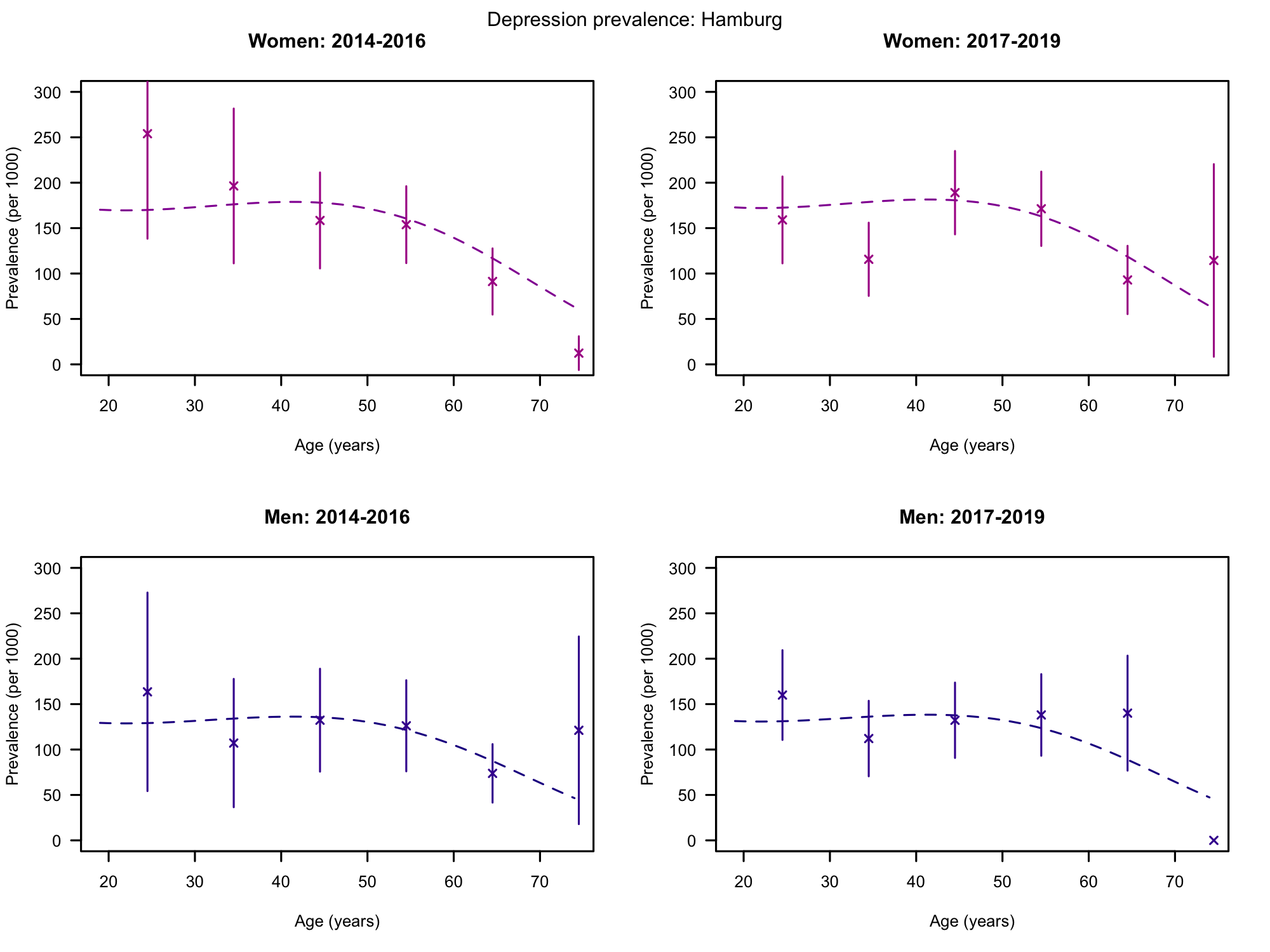


**Figure S9**. Estimated coefficients of the partial derivative $\partial p$ model for anxiety symptoms among women. Pooled estimated coefficients $\left( \beta_{0},\beta_{1},\beta_{2}, \gamma_{0},\gamma_{1},\gamma_{2} \right)$ of the partial derivative $\partial p$ model are shown for each study center. Estimates are based on 2,000 bootstrap samples. Dots indicate median point estimates, and bars represent 95% confidence intervals.


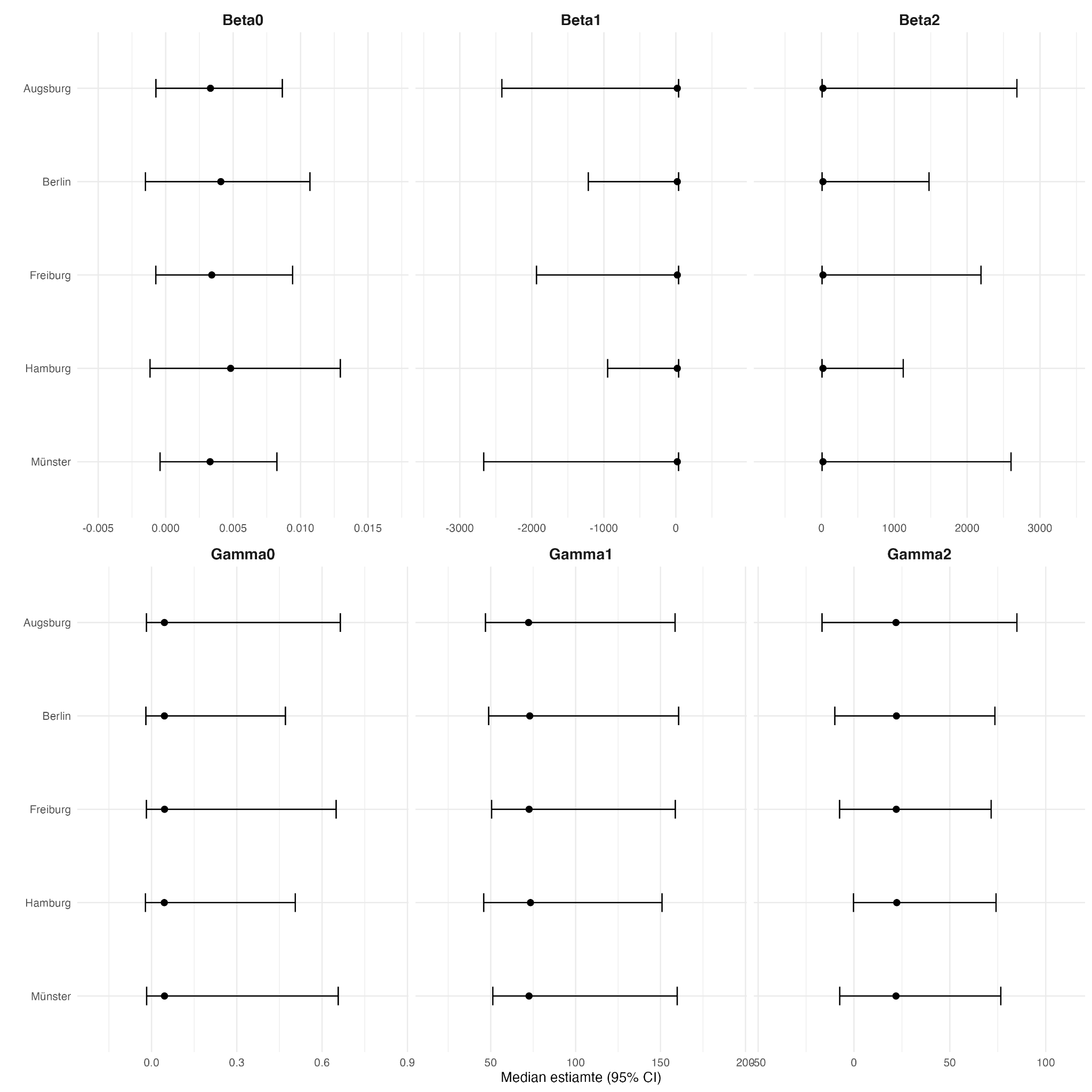


**Figure S10.** Estimated coefficients of the partial derivative $\partial p$ model for anxiety symptoms among men. Pooled estimated coefficients $\left( \beta_{0},\beta_{1},\beta_{2}, \gamma_{0},\gamma_{1},\gamma_{2} \right)$ of the partial derivative $\partial p$ model are shown for each study center. Estimates are based on 2,000 bootstrap samples. Dots indicate median point estimates, and bars represent 95% confidence intervals.


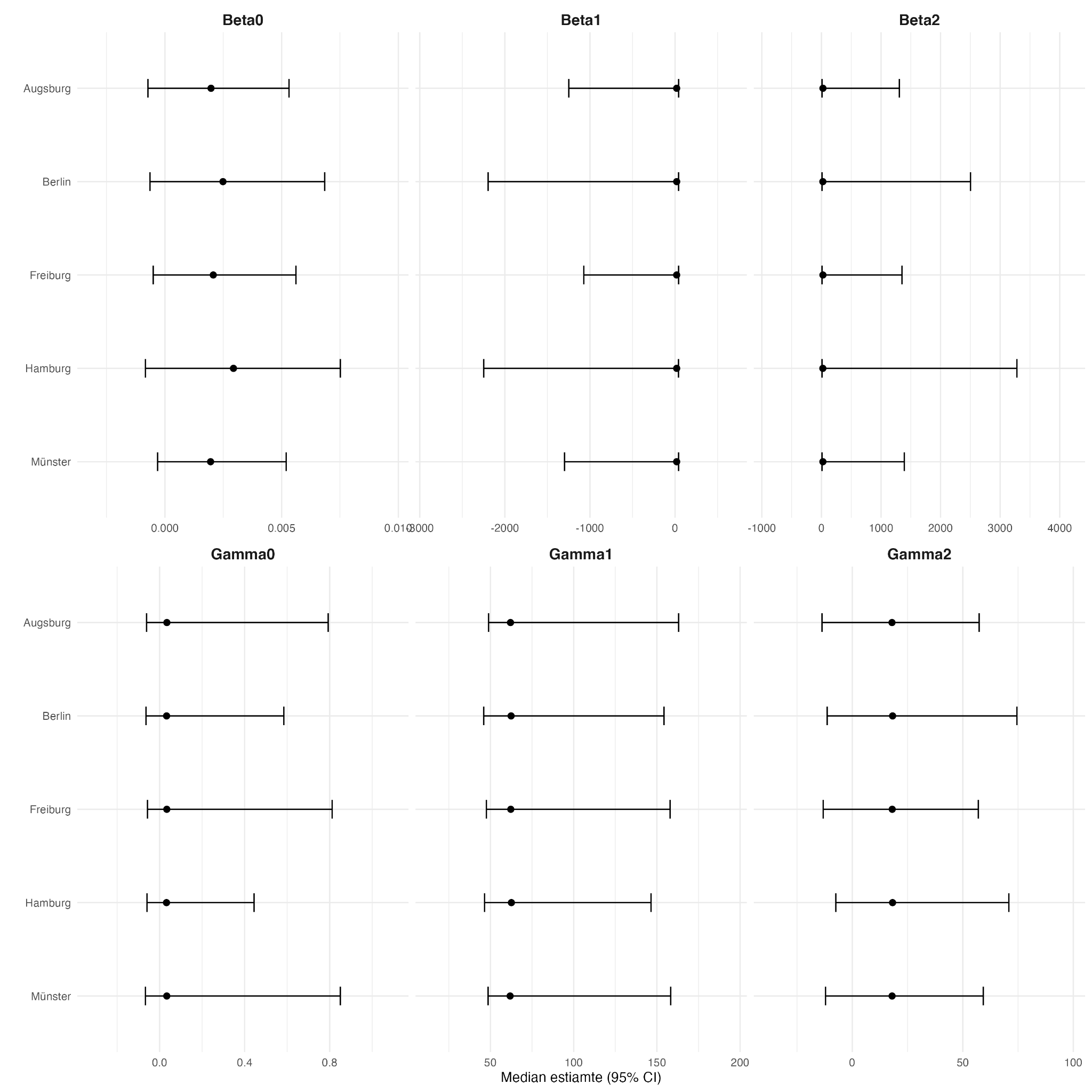


**Figure S11.** Estimated coefficients of the partial derivative $\partial p$ model for depressive symptoms among women. Pooled estimated coefficients $\left( \beta_{0},\beta_{1},\beta_{2}, \gamma_{0},\gamma_{1},\gamma_{2} \right)$ of the partial derivative $\partial p$ model are shown for each study center. Estimates are based on 2,000 bootstrap samples. Dots indicate median point estimates, and bars represent 95% confidence intervals.


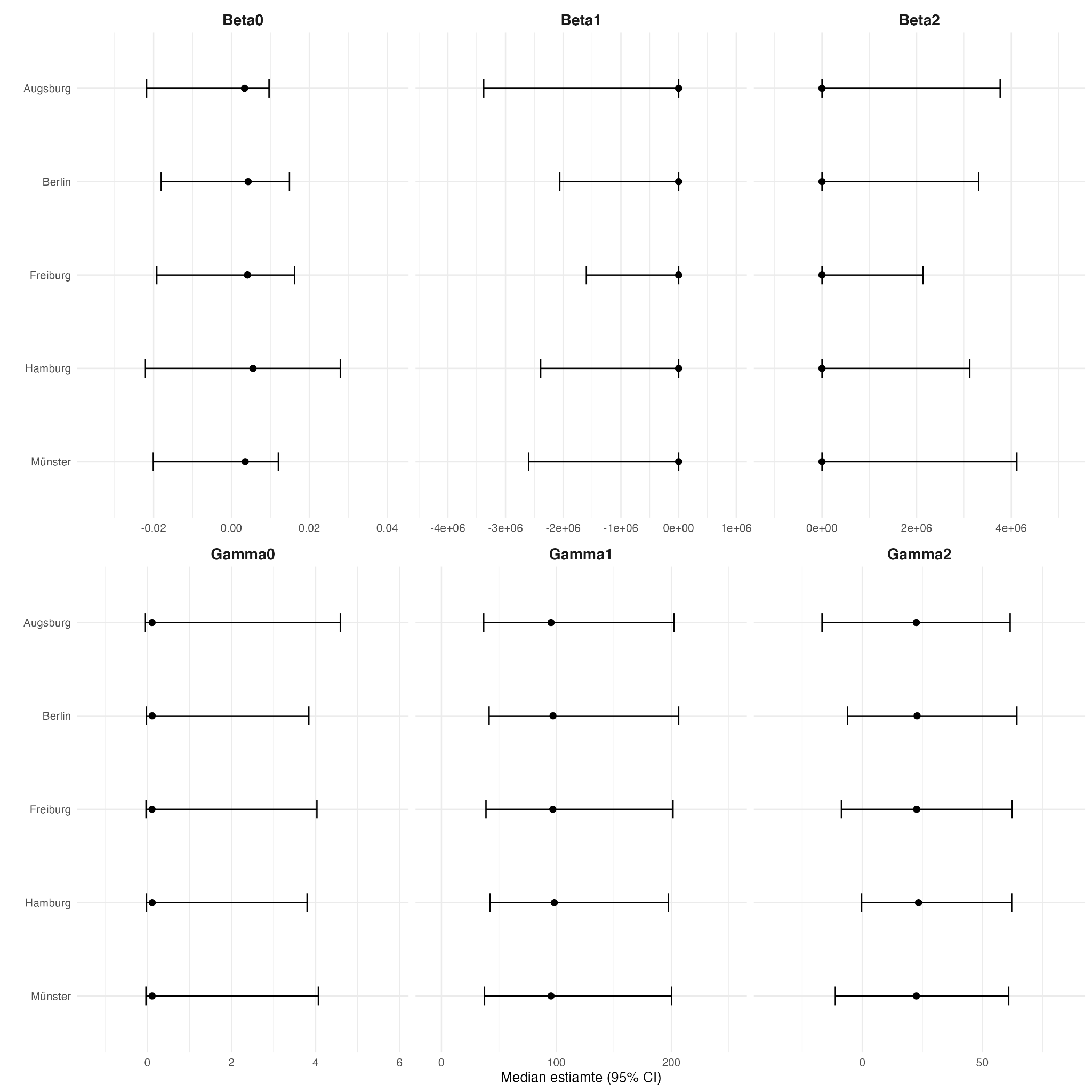


**Figure S12.** Estimated coefficients of the partial derivative $\partial p$ model for depressive symptoms among men. Pooled estimated coefficients $\left( \beta_{0},\beta_{1},\beta_{2}, \gamma_{0},\gamma_{1},\gamma_{2} \right)$ of the partial derivative $\partial p$ model are shown for each study center. Estimates are based on 2,000 bootstrap samples. Dots indicate median point estimates, and bars represent 95% confidence intervals.


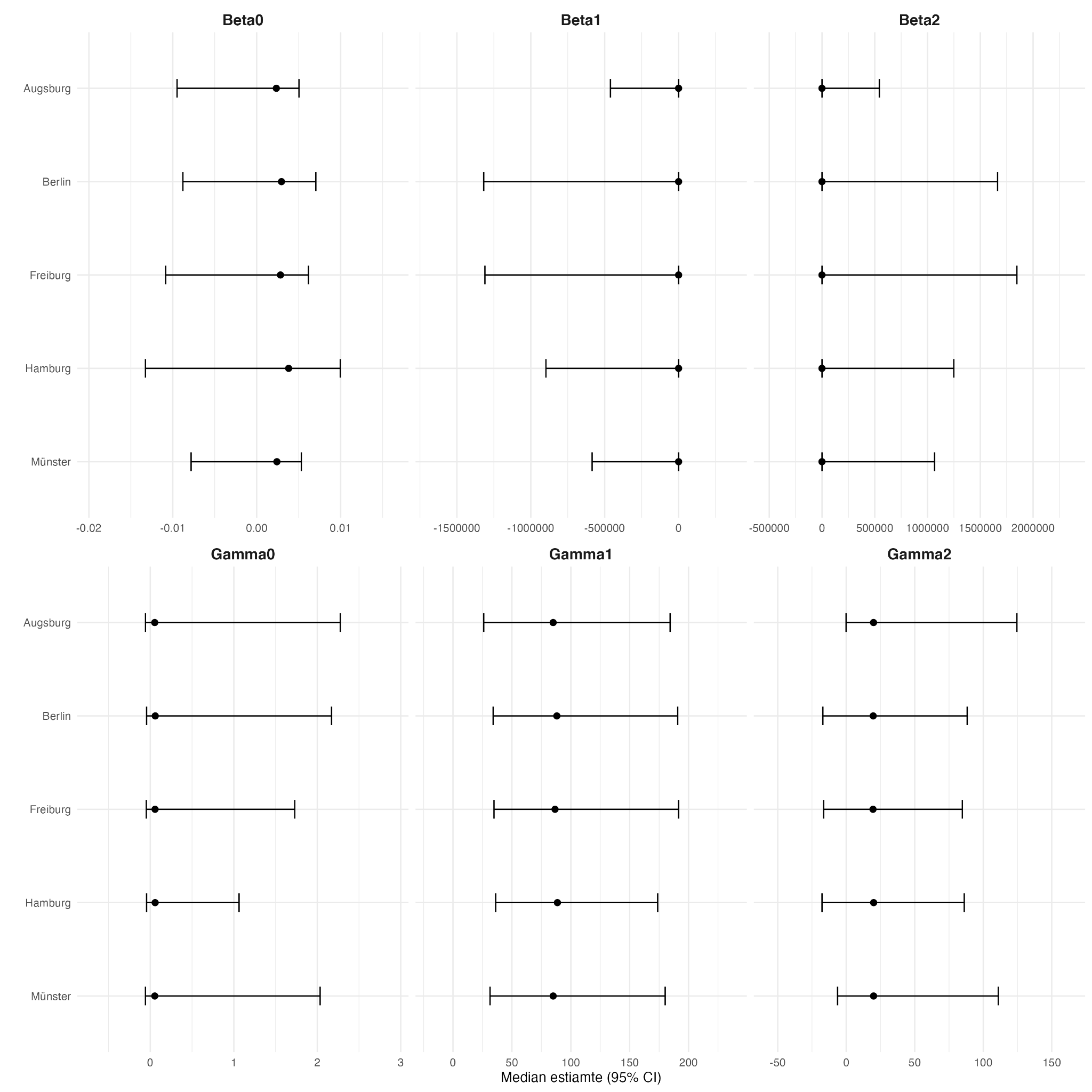


**Figure S13.** Incidence and remission rates of moderate-to-severe anxiety symptoms by age, sex, and study center under varying mortality rate ratios. Incidence rates are expressed per 1,000 person-years at risk, and remission rates per 100 person-years at risk. The solid line shows estimates based on the Danish mortality rate ratio (MRR). The dotted line shows estimates assuming the German MRR is 15% lower than that of Denmark, while the dashed line shows estimates assuming that the German MRR is 15% higher than that of Denmark.


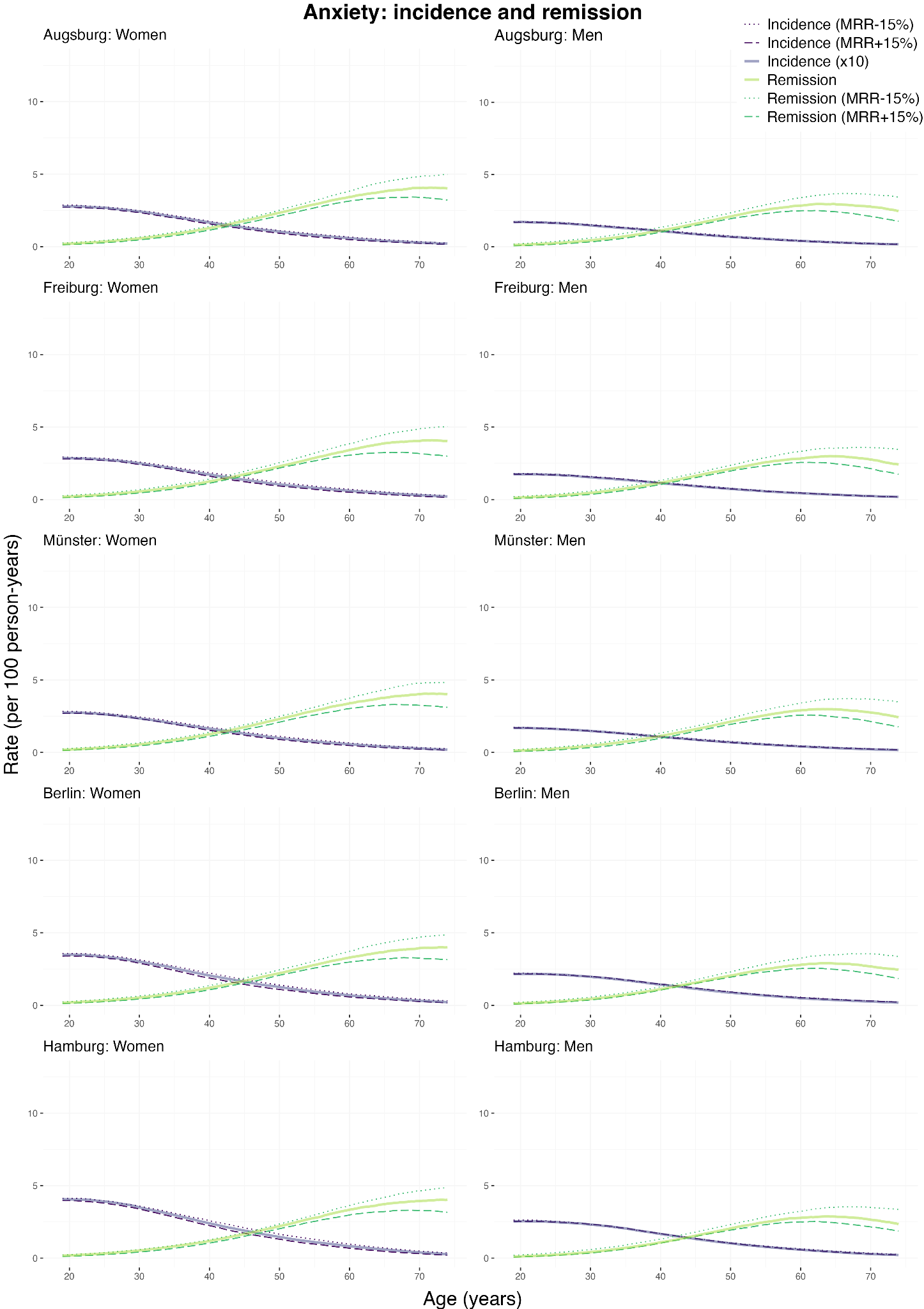


**Figure S14.** Incidence and remission rates of moderate-to-severe depressive symptoms by age, sex, and study center under varying mortality rate ratios. Incidence rates are expressed per 1,000 person-years at risk, and remission rates per 100 person-years at risk. The solid line shows estimates based on the Danish mortality rate ratio (MRR). The dotted line shows estimates assuming the German MRR is 15% lower than that of Denmark, while the dashed line shows estimates assuming that the German MRR is 15% higher than that of Denmark.


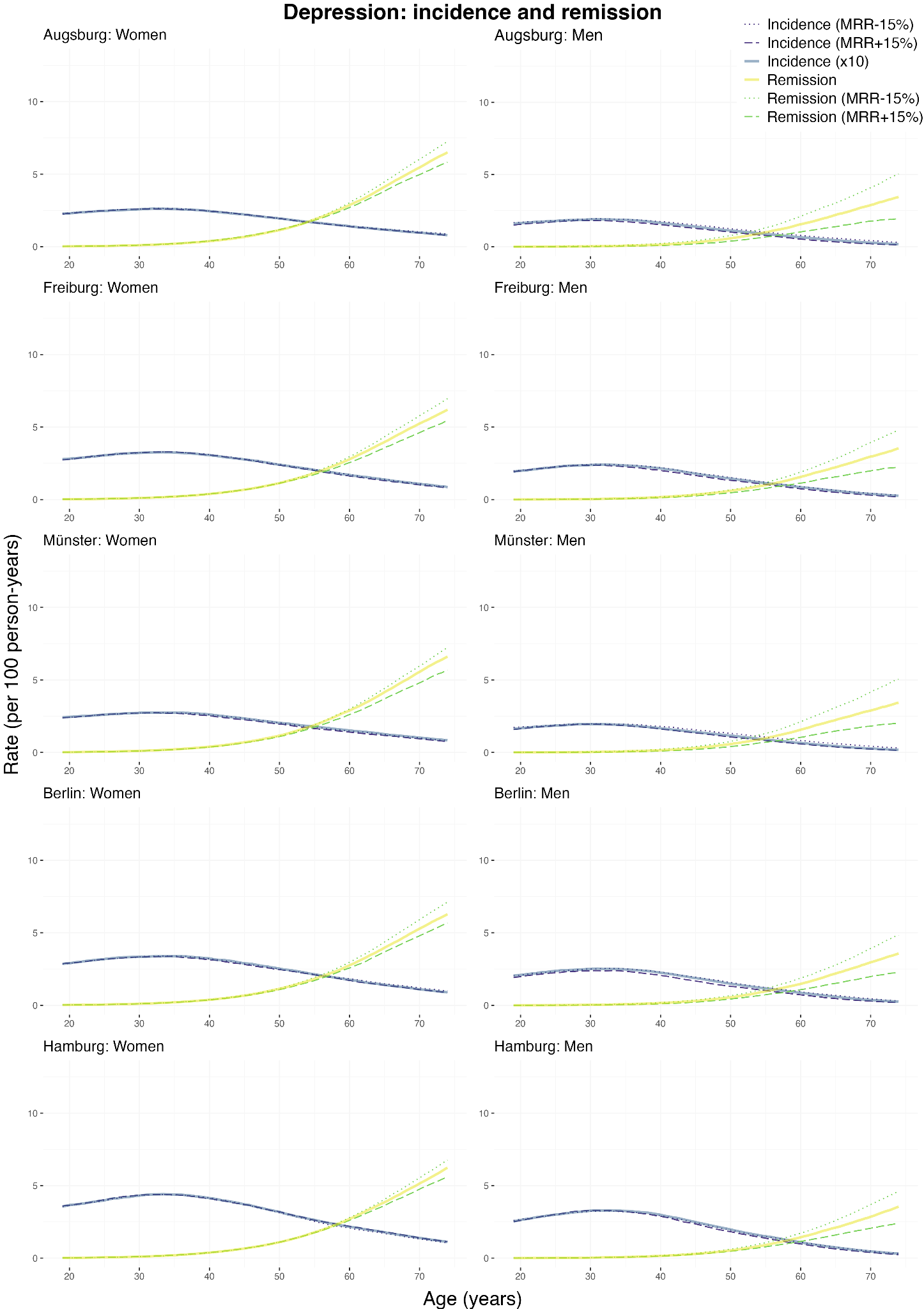

Supplement: Ito et al. supplementary material [file S0924933826101540sup001.docx]
